# In-situ muconic acid extraction reveals sugar consumption bottleneck in a xylose-utilizing *Saccharomyces cerevisiae* strain

**DOI:** 10.1186/s12934-021-01594-3

**Published:** 2021-06-07

**Authors:** Thomas Nicolaï, Quinten Deparis, María R. Foulquié-Moreno, Johan M. Thevelein

**Affiliations:** 1 Laboratory of Molecular Cell Biology, Institute of Botany and Microbiology, KU Leuven, Leuven-Heverlee, Belgium; 2 Center for Microbiology, VIB, Kasteelpark Arenberg 31, 3001 Leuven-Heverlee, Flanders Belgium; 3 NovelYeast Bv, Open Bio-Incubator, Erasmus High School, Laarbeeklaan 121, 1090 Brussels (Jette), Belgium

**Keywords:** Yeast cell factory, Muconic acid, Xylose, PDC negative, In-situ product removal, Lignocellulosic biomass, Glucose, ISPR, Pyruvate decarboxylase negative

## Abstract

**Background:**

The current shift from a fossil-resource based economy to a more sustainable, bio-based economy requires development of alternative production routes based on utilization of biomass for the many chemicals that are currently produced from petroleum. Muconic acid is an attractive platform chemical for the bio-based economy because it can be converted in chemicals with wide industrial applicability, such as adipic and terephthalic acid, and because its two double bonds offer great versatility for chemical modification.

**Results:**

We have constructed a yeast cell factory converting glucose and xylose into muconic acid without formation of ethanol. We consecutively eliminated feedback inhibition in the shikimate pathway, inserted the heterologous pathway for muconic acid biosynthesis from 3-dehydroshikimate (DHS) by co-expression of DHS dehydratase from *P. anserina*, protocatechuic acid (PCA) decarboxylase (PCAD) from *K. pneumoniae* and oxygen-consuming catechol 1,2-dioxygenase (CDO) from *C. albicans*, eliminated ethanol production by deletion of the three *PDC* genes and minimized PCA production by enhancing PCAD overexpression and production of its co-factor. The yeast pitching rate was increased to lower high biomass formation caused by the compulsory aerobic conditions. Maximal titers of 4 g/L, 4.5 g/L and 3.8 g/L muconic acid were reached with glucose, xylose, and a mixture, respectively. The use of an elevated initial sugar level, resulting in muconic acid titers above 2.5 g/L, caused stuck fermentations with incomplete utilization of the sugar. Application of polypropylene glycol 4000 (PPG) as solvent for in situ product removal during the fermentation shows that this is not due to toxicity by the muconic acid produced.

**Conclusions:**

This work has developed an industrial yeast strain able to produce muconic acid from glucose and also with great efficiency from xylose, without any ethanol production, minimal production of PCA and reaching the highest titers in batch fermentation reported up to now. Utilization of higher sugar levels remained conspicuously incomplete. Since this was not due to product inhibition by muconic acid or to loss of viability, an unknown, possibly metabolic bottleneck apparently arises during muconic acid fermentation with high sugar levels and blocks further sugar utilization.

**Supplementary Information:**

The online version contains supplementary material available at 10.1186/s12934-021-01594-3.

## Background

**Fig. 1:**
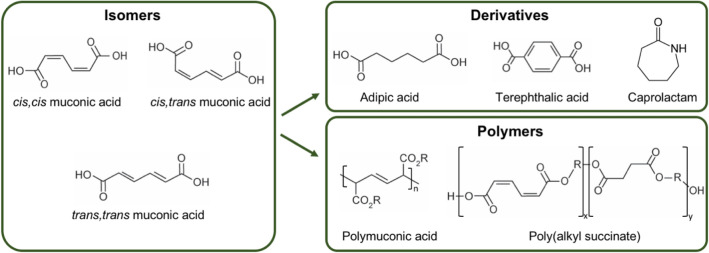
Chemical structures of muconic acid isomers, derivatives and polymers

The current shift from a fossil-based economy to a more sustainable, bio-based economy requires development of alternative production routes not only for fuels but also for the many chemicals that are currently produced from petroleum [1–3]. Over the last decade there has been an increased interest in microbial production of muconic acid. Muconic acid is an attractive molecule for the bio-based economy because it can be converted into important platform chemicals, such as adipic acid and terephthalic acid [4]. It can also be used as a monomer in the production of heteropolymers [5] or homopolymers [6, 7] while retaining its double bonds, which allows for straightforward further chemical modification (Fig. 1).

Ideally, bio-based platform chemicals, such as muconic acid, are produced from second-generation substrates that are plentiful and cheap [8]. Lignocellulosic biomass is an excellent choice, but its use poses several challenges [9, 10]. One major challenge is the efficient utilization of xylose [11, 12]. Two studies have reported the conversion of xylose into muconic acid, one using an *E. coli* co-culture [13] and the other an engineered *S. cerevisiae* lab strain [14]. Neither of these can be implemented in industrial practice due to their lack of robustness and inhibitor tolerance [9].

The most widely used de novo biosynthesis pathway for muconic acid starts with dehydroshikimate (DHS) as intermediate of the shikimate pathway. DHS is converted to protocatechuic acid (PCA), catechol and muconic acid, by combined expression of DHS dehydratase (DHSD), protocatechuic acid decarboxylase (PCAD) and catechol 1,2-dioxygenase (CDO). By further increasing availability of the shikimate pathway substrates, erythrose 4-phosphate (E4P) and phosphoenolpyruvate (PEP), and also enhancing DHS availability, Bui et al. obtained the highest muconic acid titer of 59 g/L from glucose using *E. coli* as the host organism [15]. Although currently the highest reported muconic acid yields were obtained with bacteria as host organisms, they are not preferred for industrial production because of contamination risks and their sensitivity to low pH, which complicates downstream processing of the muconic acid produced [16]. Industrial strains of the yeast *S. cerevisiae* are considered a more preferred alternative because of their high tolerance to low pH, general robustness and high stress tolerance in industrial conditions.

The first study implementing *S. cerevisiae* as a host organism reported a very low titer of 1.56 mg/L and a yield of 0.78 mg/g [17]. In subsequent studies two modifications were applied to enhance titer and yield [18]. The feedback insensitive 3-deoxy-d-arabino-heptolusonate-7-phosphate (DAHP) synthase enzymes, *ARO3*^*K222L*^ and *ARO4*^*K229L*^ were expressed to increase flux into the shikimate pathway [19–23], as well as transketolase *TKL1* to enhance E4P availability [20, 21, 23–25].

Other studies have used various heterologous genes to achieve higher muconic acid titers [21–25]. The PCAD enzyme received particular attention because of the high PCA accumulation. In general the *K. pneumoniae* PCAD enzyme has been preferred and is encoded by the native BCD cluster. While the C isoform (*aroY-*C) encodes the actual PCAD enzyme, the B isoform (*aroY-*B) has a supportive function by production of the prenylated flavin mononucleotide (prFMN) cofactor [26]. Another PCAD homologue (*aroY-C*^*iso*^) was shown to be less oxygen sensitive displaying higher activity under oxygen-limited conditions [17, 27]. The gene *PAD1* in *S. cerevisiae* is a paralog to *aroY-B* and its overexpression led to increased PCAD activity [28]. Increasing the copy number of co-expressed *aroY-B* and *aroY-C*^*iso*^ enhanced PCAD activity and muconic acid production, confirming the rate-limiting character of PCAD [25, 29]. Alternative prFMN-independent PCAD enzymes were shown to be superior to the co-expression of *aroY-B* and *aroY-C*^*iso*^ in yeast strains that do not express *PAD1* [20, 30], but it remains unclear whether they support higher PCAD activity than the co-expression of *PAD1* and *aroY-C*^*iso*^. Recently, Wang et al. expressed a heterologous muconic acid production pathway in *S. cerevisiae* and enhanced the performance of the strain through biosensor-aided and subsequent rational genomic engineering approaches. This study also showed that muconic acid was toxic at concentrations of 5 g/L and higher when the pH was not controlled. Ethanol production was lowered by optimizing the feed solution and feeding rate in a controlled fed-batch fermentation. These optimizations combined with maintenance of the pH at 6 to minimize toxicity, led to a titer of 20.8 g/L and a yield of 66.2 mg/g muconic acid, which is the highest titer and yield for muconic acid production with *S. cerevisiae* reported to date.

Up until now, none of the yeast strains used for muconic acid production completely lacked ethanol production, likely a major cause of the low yields obtained. Ethanol production can be eliminated by deleting the three pyruvate decarboxylase (PDC) encoding genes *PDC1*, *PDC5* and *PDC6*. However, this severely compromises growth and metabolism of the yeast because of shortage in cytosolic acetyl-CoA [31, 32]. This problem can be addressed to some extent by supplying a C2 compound such as ethanol in the medium, or by a partial internal deletion of the *MTH1* gene [33–38]. However, neither approach overcomes the problem completely and elimination of ethanol production to enhance yield thus remains challenging.

A universal stress factor for cell factories is toxicity of the end-product, because it has to be produced at a high titer to ensure efficient downstream processing for the economic viability of the industrial process. While increasing the pH of the fermentation medium results in lower muconic acid toxicity [39], it adds complexity to the muconic acid recovery process and compromises economic feasibility. One way to circumvent this problem is in-situ product removal (ISPR) of muconic acid during the production process. Various methodologies exist for extracting carboxylic acids [40] and more specifically muconic acid [41–45]. Although the suggested solvents are supposedly biocompatible, there are no published results on the use of these solvents in combination with host organisms producing muconic acid.

In this work we have engineered an industrial second-generation yeast strain with efficient xylose consumption and high inhibitor tolerance for muconic acid production. We eliminated for the first time ethanol production completely, strongly minimized production of the PCA intermediate and optimized the strain for maximal titer, productivity and yield. With this strain we produced the highest reported titer of muconic acid in batch fermentations to date. To overcome stuck fermentations when using a high initial sugar level, presumed to be due to muconic acid toxicity at low medium pH, we developed ISPR with polypropylene glycol 4000 (PPG) as solvent. This surprisingly revealed an unexpected, possibly metabolic bottleneck in utilization of high sugar levels arising during muconic acid production and unrelated to its toxicity.

## Materials and methods

### Yeast strains and plasmids

The yeast strains and plasmids used and constructed in this work are shown in Tables 1 and 2, respectively.

**Table 1:** Strains used and constructed in this study

Yeast strains	Description	Source
	Genotype	
W303	*MATα can1-100 ade2-1 his3-11,15 leu2-3 trp1-1 ura3-1*	MCB, KU Leuven
GSE16-T18HAA1	Industrial 2G xylose-fermenting and inhibitor-tolerant strain GSE16-T18 with C.1517 G>A mutation in both copies of *HAA1*	[46]
TN7	GSE16-T18HAA1 *aro3∆ ARO4*^*K229L*^	This study
TN8	TN7 *MTH1*^*∆M41*-*T78*^	This study
TN9	TN8 *pdc1::*MApw MApw being TDH3p - *HQD2*_*Ca*_ - ADH1t, ADH1p - *aroY-C*^*iso*^ - ADH2t, TEF1p - *aroZ*_*Pan*_ - CYC1t	This study
TN10	TN9 IS2.1:: FBA1p - *PAD1* - ADH1t IS2.1, integrative site NC_001134:g.140631^14632 (yeast 2015)	This study
TN5	TN10 *pdc6::*Mapw Mapw being TDH3p - *HQD2*_*Ca*_ - ADH1t, ADH1p - *aroY-C*^*iso*^ - ADH2t, TEF1p - *aroZ*_*Pan*_ - CYC1t	This study
TN6	TN5 *pdc5*::Mapw Mapw being TDH3p - *HQD2*_*Ca*_ - ADH1t, ADH1p - *aroY-C*^*iso*^ - ADH2t, TEF1p - *aroZ*_*Pan*_ - CYC1t TN6-1, TN6-2, TN6-3, TN6-4 and TN6-5 are independent transformants	This study
TN14	TN6-1 IS16.2:: PGK1p - *AGDC1* - TPS1t IS16.2, integrative site NC_001148:g.621736^621737 (yeast 2015)	This study [20]
TN15	TN6-1 IS16.2:: PGK1p - *TaGDC1* - TPS1t IS16.2, integrative site NC_001148:g.621736^621737 (yeast 2015)	This study [20]
TN16	TN6-1 *aroY-C*^*iso*^ (WT) *aroY-C*^*iso, I218V*^ in TN6-1 at *PDC5* locus to wild type *aroY-C*^*iso*^	This study
TN22	TN6-1 IS16.2:: PGK1p - *aroY-C*^*iso*^ - TKL1t IS16.2, integrative site NC_001148:g.621736^621737 (yeast 2015)	This study

**Table 2:** Plasmids used and constructed in this study

Plasmid	Description	Source
Generic plasmids and plasmid backbones		
pJET1.2-B-KanMX-P	pMB1 *ori* (*E. coli*) Backbone for amplification of KanMX marker flanked by attB and attP (attB/P)	MCB, KU Leuven
pTOPO-G1-NAT-G1	pMB1 *ori* (*E. coli*) Backbone for amplification of NatMX marker flanked by attB/P and G1 recognition site	MCB, KU Leuven
PhiC31NAT	ColE1 *ori* (*E. coli*) and 2 micron *ori* (*S. cerevisiae*, multi-copy*)*, encodes PhiC31 integrase and NatMX marker	MCB, KU Leuven [47, 47]
p426KanMX	pMB1 *ori* (*E. coli*) and 2 micron *ori* (*S. cerevisiae*, multi-copy*)*, KanMX marker Backbone for construction of donor DNA	MCB, KU Leuven
p426hph	pMB1 *ori* (*E. coli*) and 2 micron *ori* (*S. cerevisiae*, multi-copy*)*, hph marker Backbone for construction of Mapw and donor DNA	MCB, KU Leuven
pBEVYhph	ColE1 *ori* (*E. coli*) and 2 micron *ori* (*S. cerevisiae*, multi-copy*)*, hph marker Backbone for construction of Mapw	MCB, KU Leuven
pTEF-Cas9-KanMX (p51)	pBR322 *ori* (*E. coli*) and CEN *ori* (single copy), vector backbone p414-TEF1p-Cas9-CYC1t with auxotrophic marker replaced by KanMX	MCB, KU Leuven
pgRNA-uni-NAT (p59)	pBR322 *ori* (*E. coli*) and 2 micron *ori* (*S. cerevisiae*, multi-copy) gRNA plasmid backbone with NatMX marker	MCB, KU Leuven
pgRNA-uni-hph (p58)	pBR322 *ori* (*E. coli*) and 2 micron *ori* (*S. cerevisiae*, multi-copy) gRNA plasmid backbone with hph marker	MCB, KU Leuven
p426hph-IS2.1	Multi copy, homologous regions for IS2.1 with hph marker	MCB, KU Leuven
p426hph-IS16.2	p426hph backbone with homologous regions for IS16.2	MCB, KU Leuven
Guide RNA plasmids		
pgRNA-uni-hph-G1-G2	P58 backbone with gRNA sequence targeting G1 (GGCTGATTTTCGCAGTTCGG) and G2 (GGATGAGAATCTGACAAAGGG)	MCB, KU Leuven
p58-*ARO4*	p58 backbone with gRNA targeting *ARO4* (TTCATGGGTGTTACTAAGCA)	This study
p58-*MTH1*	p58 backbone with gRNA sequence targeting *MTH1* (CTAGCTCTATCAGTGTACTC)	This study
p58-*PDC5*	p58 backbone with two gRNA sequences targeting *PDC5* (AGCATCCAACAATTTTTGCA and GATAAGCTTTATGAAGTCAA)	This study
p58-*PDC6*	p58 backbone with two gRNA sequences targeting *PDC6* (CTATCGAAAAGCTGATTCAT and GCTGATTTGATCCTTTCGGT)	This study
p59-*aroY-C*^*iso,I218V*^	p59 backbone with gRNA sequence targeting *aroY-C*^*iso,I218V*^ (TTAGATCCCGCTATCTACGT)	This study
p59-attL	Multi copy, gRNA for attL site	This study
p59-IS2.1	p59 backbone with gRNA sequence targeting the IS2.1 site (ATCAACCACAGTGAACGCCG)	This study
p59-IS16.2	p59 backbone with gRNA sequence targeting the IS16.2 site (GTAGAATAAGTGTTTCGGAT)	This study
Plasmids containing expression cassettes		
p426KanMX-*PAD1*	p426KanMX backbone with the expression cassette FBA1p; *PAD1*; ADH2t	This study
p426hph-IS2.1-*PAD1*	p426hph-IS2.1 backbone with the expression cassette FBA1p; *PAD1*; ADH2t flanked by the homologous regions for genomic integration at IS2.1	This study
p426KanMX-*aroY-B*	p426KanMX backbone with the expression cassette FBA1p; *aroY-B*; ADH2t	This study
p426KanMX-DHSD	p426KanMX backbone with the expression cassette TEF1p; *aroZ*_*pan*_; CYC1t	This study
pBEVYhph-CDO-PCAD(aroY-C*iso*)	pBEVYhph backbone with the expression cassettes TDH3p - *HQD2*_*Ca*_ - ADH1t, ADH1p - *aroY-C*^*iso*^ - ADH2t Together with p426KanMX-DHSD constitutes pMApw(aroY-C^iso^)	This study
pBEVYhph-CDO-PCAD(aroY-C*iso*)-DHSD (pMApw)	pBEVYhph backbone with the expression cassettes TDH3p - *HQD2*_*Ca*_ - ADH1t, ADH1p - *aroY-C*^*iso*^ - ADH2t, TEF1p - *aroZ*_*pan*_ - CYC1t Contains the muconic acid pathway (MApw)	This study
pMApw-*PDC1*	pMApw backbone with homologous regions for genomic integration at the *PDC1* locus	This study
pMApw-*PDC5*	pMApw backbone with homologous regions for genomic integration at the *PDC5* locus	This study
pMApw-*PDC6*	pMApw backbone with homologous regions for genomic integration at the *PDC6* locus	This study
p426hph-IS16.2-*AGDC1*	p426hph-IS16.2 backbone with the expression cassette PGK1p - *AGDC1* - TPS1t flanked by the homologous regions for genomic integration at IS16.2	This study
p426hph-IS16.2-*TaGDC1*	p426hph-IS16.2 backbone with the expression cassette PGK1p - Ta*GDC1* - TPS1t flanked by the homologous regions for genomic integration at IS16.2	This study
p426hph-IS16.2-*aroY-C*^*iso*^	p426hph-IS16.2 backbone with the expression cassette PGK1p - *aroY-C*^*iso*^ - TPS1t flanked by the homologous regions for genomic integration at IS16.2	This study
p426hph-IS16.3-*ARO3*^*K222L*^*-ARO4*^*K229L*^	p426hph-IS16.3 backbone with the expression cassettes PGK1p - *ARO3*^*K222L*^ - TPS1t, TEF1p; *ARO4*^*K229L*^ - ELO2t flanked by the homologous regions for genomic integration at IS16.3	This study

### Growth media and culturing conditions

Propagation of yeast cells was performed in YPD medium containing 1% yeast extract, 2% bacteriological peptone and 2% glucose at 30 °C in a rotary shaker (200 rpm). For propagation of *pdc*-negative strains, glucose was replaced by 2% glycerol and 2% ethanol as carbon source. In order to have sufficient aeration during growth of *pdc-*negative strains, propagation was performed in shake flasks (100 mL or 300 mL) under constant shaking (200 rpm). Media were supplemented with 1.5% bacto™ agar to obtain solid nutrient agar plates. Transformants were selected on solid agar plates supplemented with either one or a combination of the following antibiotics: 200 mg/L geneticin, 300 mg/L hygromycin B or 200 mg/L nourseothricin. In case of *pdc*-negative strains, only 20% of the abovementioned amounts of antibiotics was used. For storage of strains for longer periods of time, 30% glycerol was added to YP medium in sterile screw-cap tubes which were stored at − 80 °C. Cells were plated out and incubated at 30 °C when taken out of the stock. Maintenance and selection of plasmids in *E. coli* (TOP10) was performed in lysogeny broth (LB) medium supplemented with 100 mg/L ampicillin or 50 mg/L kanamycin. Solid LB plates contained 1.5% bacto™ agar.

### Genomic DNA and plasmid extraction, PCR

Genomic DNA of yeast cells was extracted by phenol/chloroform/isoamyl-alcohol (25:24:1) and precipitated with ethanol. PCR was performed with low-fidelity Standard Taq or TaqE polymerase to test for positive transformants or with high-fidelity polymerase Q5 for sequencing and cloning.

Plasmids were extracted from *E. coli* using the commercial Nucleospin Plasmid EasyPure kit (Macherey-Nagel) after which digestion with relevant restriction enzymes was performed. Cloned plasmids were transformed in *E. coli* and transformants selected on LB agar plates containing the appropriate antibiotic. Transformants were checked by colony-PCR for presence of the correct construct using low-fidelity Standard Taq or TaqE polymerase. Positive transformants were grown overnight in liquid LB medium, supplemented with the appropriate antibiotic. Plasmids were submitted for Sanger sequencing at the Genetic Service Facility of the VIB.

### Gene synthesis and cloning

The ORF of the following heterologous genes was codon optimized and ordered at IDT: DHSD from *Podospora anserina* (*aroZ*_*Pan*_*/*PA_5_5120; GeneBank Accession number CAD60599), CDO from *Candida albicans* (HQD2_Ca_; GeneBank Accession number KHC86777), PCAD from *Klebsiella pneumoniae* (*aroY-C*^*iso*^; GeneBank Accession number AB479384), *Arxula adeninivorans* (*AGDC1*; GeneBank Accession number SJN60119.1) and *Talaromyces atroroseus* (*TaGDC1*, GeneBank Accession number XP 020118381.1), and flavin prenyltransferase from *K. pneumoniae* (*aroY-B*, GeneBank Accession number AAY57854). Using the high-fidelity polymerase Q5 (New England Biolabs) the genes obtained were PCR-amplified with a low cycle number to prevent accidental mutations. Next, the amplicons were purified with the commercial Wizard SV Gel and PCR clean-up (Promega) kit and cloned into the respective vectors using Gibson assembly master mix (New Englands Biolabs). The ORF of the *PAD1* gene was amplified from the genome of the W303 lab strain.

### Strain construction

Yeast transformation was performed either by Lithium-acetate protocol or electroporation [48, 49]. Deletion of *ARO3* was performed with a KanMX marker, flanked with attB and attP recombination sites. Removal of the integrated KanMX marker in the positive transformants was achieved by transformation of the PhiC31 integrase vector, which recombines attB and attP, leaving an attL scar. The remaining genomic adjustments were carried out with the CRISPR/Cas9 genome editing tool. Yeast cells were transformed with the p51 plasmid, carrying the Cas9 enzyme, and precultured for a second transformation in YP media with the appropriate carbon source and geneticin. During the second transformation, gRNA plasmid and linear donor DNA (amplified from the donor DNA backbone with high-fidelity Q5 polymerase) were transformed into the yeast cells and the transformants were selected for presence of both Cas9 and gRNA plasmids. The *PDC1* gene was replaced by a step-wise approach in which the ORF was first replaced by a NatMX marker, flanked with G1 gRNA recognition sites that were targeted in a subsequent transformation with the G1 gRNA. Other genomic adjustments and knock-ins were conducted by a one-step approach, directly targeting and replacing genomic DNA. Plasmid loss was achieved by 3× serial transfer in YP medium, after which single cells were isolated and spotted on solid agar medium containing relevant antibiotics to screen for loss of resistance.

### Muconic acid fermentations and HPLC analysis

Yeast cells were propagated in YP medium with the appropriate carbon source till stationary phase. Cells were washed with sterile water and inoculated at different OD_600_ levels, as indicated, in 300 mL shake flasks with a total of 40 mL YP medium. Technical duplicates or biological triplicates were used for each strain, with the standard deviation indicated. Samples for pH measurements and metabolite analysis were taken at various time points. For HPLC analysis, the supernatant was separated from the cells by centrifugation (12,000 rpm, 2 min) and analysed with a Biorad Aminex HPX 300 × 7.8 mm column. From the supernatant, 10 μL was injected in a 5 mM H_2_SO_4_ mobile phase with a flow rate of 0.65 mL/min, while the column temperature was maintained at 60 °C. Protocatechuic acid, catechol and muconic acid were detected at 270 nm using the UV detector (SPD-20A, Shimadzu). Glucose, xylose and ethanol were quantified using the RID detector (RID-20A, Shimadzu). In both cases standard curves were used.

### Whole genome sequence analysis

Genomic DNA was extracted using the MasterPure Yeast DNA purification kit (Epicentre, Lucigen). Strains TN5 (Samplen220) and TN6-1 (Samplen221) were sent to BGI (China) for next-generation sequencing with Illumina (HiSeq2500) using a 500 bp insert library and pair-end reads of 125 bp. Strains TN6-2 (Samplen267), TN6-3 (Samplen268), TN6-4 (Samplen269) and TN6-5 (Samplen270) were sent to Eurofins Genomics (Germany) for next-generation sequencing with Illumina (HiSeq2500) using a 500 bp insert library and pair-end reads of 150 bp. The reads were mapped against the S288c reference genome (R64-2,1; SGD) using bowtie 2 [50]. Coverage of the TN5 and the different TN6 strains was determined using samtools [51] and compared to the GSE16-T18HAA1 strain using NGSEP [52]. Variants were called using NGSEP and SNP frequency was determined using in-house scripts. The data have been deposited with links to the BioProject accession number PRJNA699459 in the NCBI BioProject database (https://www.ncbi.nlm.nih.giv/bioproject/).

### Determination of distribution coefficient (Kd) for PPG

To represent the fermentation conditions, a YPD medium was spiked with muconic acid and adjusted to a pH of 4.25 ± 0.05. Then 20% (v/v) of the solvent was added. The mixtures were shaken vigorously at 30 °C for 30 min to reach extraction equilibrium. Next, the samples were centrifuged (14,000 rpm, 3 min) and the concentration of muconic acid in the aqueous phase was determined by HPLC analysis. Using the equation below, where c_org_ and c_aq_ are the concentrations of muconic acid in the organic phase and in the aqueous phase, respectively, we calculated a distribution coefficient (Kd) of 5.78 for PPG.

$$Kd = \frac{{c_{org} }}{{c_{aq} }}$$

The c_org_ was calculated based on the concentration of muconic acid in the aqueous phase before and after extraction, while taking into account the applied volumetric solvent ratio of 20% (v/v).

## Results

### Construction of a xylose-utilizing *pdc- S. cerevisiae* strain with efficient muconic acid production

**Fig. 2:**
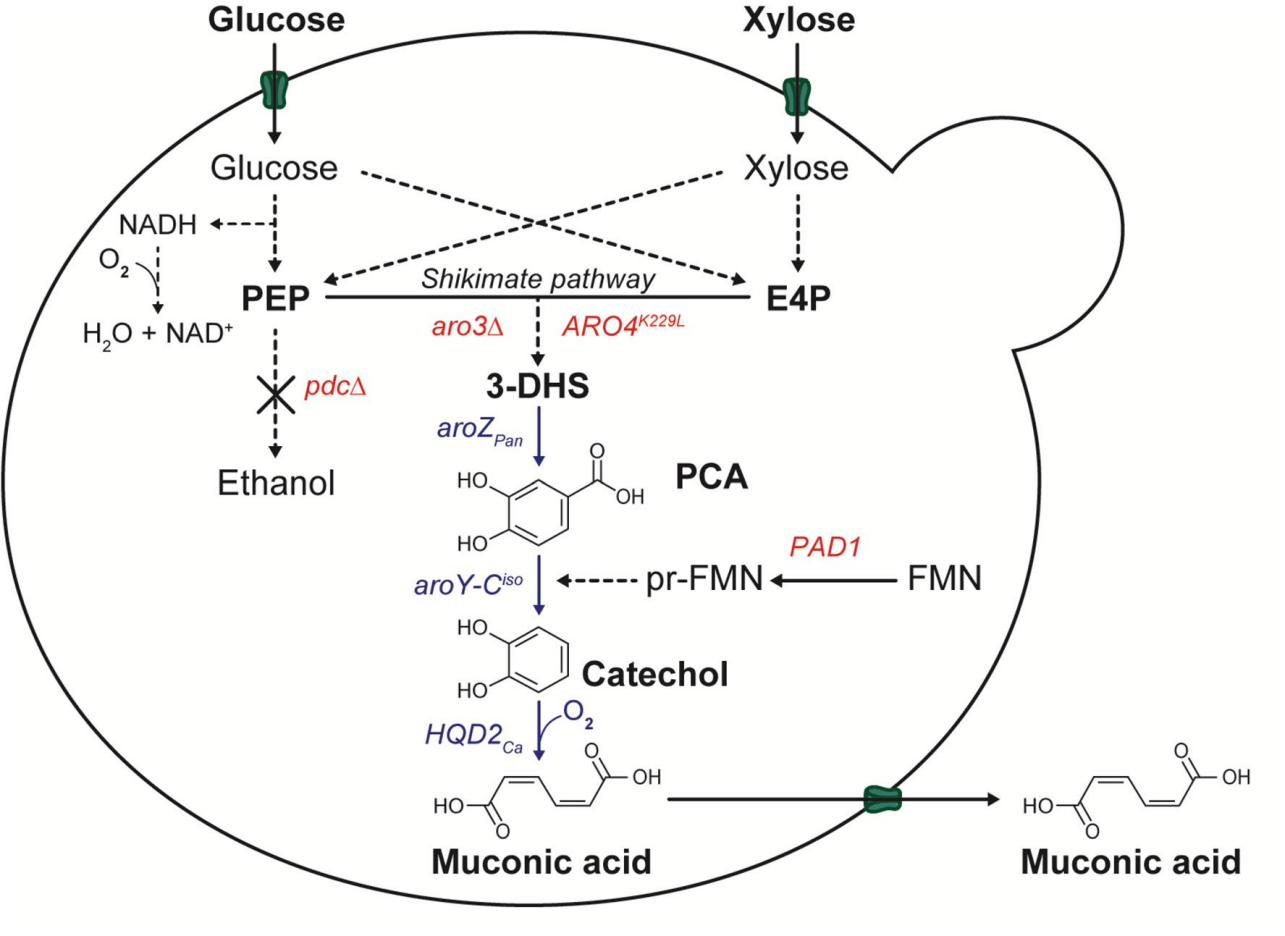
Pathways used to produce muconic acid from glucose and xylose. Glucose and xylose are converted after uptake into phosphoenolpyruvate (PEP) and erythrose-4-phosphate (E4P) by the glycolytic and pentose-phosphate pathways. The two compounds are combined to enter the shikimate pathway and produce 3-dihydroshikimate (DHS), which is then converted using the bacterial heterologous pathway for muconic acid biosynthesis (MApw) into protocatechuic acid (PCA), catechol and muconic acid. The MApw consists of a DHS dehydratase from *Podospora anserina *(aroZ_Pan_), PCA decarboxylase (PCAD) from *K. pneumoniae* (*aroY-C*^*iso*^) and catechol dioxygenase (CDO) from *C. albicans* (*HQD2*_*Ca*_). Feedback inhibition of aromatic amino acids acting on Aro3 and Aro4, has been relieved by deletion of Aro3 and site-specific mutagenesis of Aro4 (*ARO4*^*K229L*^). Overexpression of *PAD1* stimulates production of prenylated flavine mononucleotide (prFMN), a co-factor of the protocatechuic acid decarboxylase (PCAD) enzyme encoded by *aroY-C*^*iso*^. To further enhance muconic acid production, the synthesis of ethanol was abolished by deletion of the pyruvase decarboxylase (*PDC*) genes (*pdcΔ*). Muconic acid is exported from the cells by one or more unknown carriers

To construct an industrially relevant yeast strain for muconic acid production from lignocellulosic biomass, we used the diploid *S. cerevisiae* second-generation bioethanol strain GSE16-T18 HAA1 as a host strain [46]. First, we focused on improving the flux in the central shikimate pathway, used for production of aromatic amino acids, to improve the availability of the DHS intermediate that will serve as substrate for the heterologous muconic acid pathway. For that purpose, we deleted *ARO3* and introduced the point mutation K229L in *ARO4* to overcome the feedback inhibition in the shikimate pathway (Fig. 2), which resulted in strain TN7.

Next, we constructed a plasmid with the heterologous pathway for muconic acid production (MApw) by co-expression of three codon-optimized heterologous enzymes: DHSD from *P. anserina* (*aroZ*_*Pan*_/PA_5_5120), PCAD from *K. pneumoniae* (*aroY-C*^*iso*^) and CDO from *C. albicans* (*HQD2*_*Ca*_) under control of the *TEF1, ADH1* and *TDH3* constitutive promoter, respectively. We introduced the plasmid expressing the MApw into the TN7 strain, which resulted in high production of the PCA intermediate and a transient production of minor levels of muconic acid due to its consumption under aerobic conditions (Additional file 1).

Next we eliminated ethanol production by deleting both copies of the *PDC1, PDC5, PDC6* genes. For that purpose, we first deleted part of the *MTH1* gene (*MTH1*^*ΔM41*-*T78*^), which is known to alleviate glucose repression and restore growth on glucose in *pdc-* strains [35, 38]. This resulted in strain TN8 (TN7 *MTH1*^*ΔM41*-*T78*^).

Next, we integrated the MApw in the genome at the *PDC1* locus, replacing the two copies of the *PDC1* gene, yielding strain TN9 (TN8 *pdc1::*MApw/*pdc1::*MApw). Since this strain still produced large amounts of PCA and only minor amounts of muconic acid (Additional file 2), we increased PCAD activity by expression of *PAD1* or *aroY-B* on a multi-copy plasmid. Since expression of *PAD1* resulted in a higher muconic acid level (Additional file 3), we next introduced two copies of *PAD1* in the genome under control of the *FBA1* promoter, resulting in the strain TN10 (TN9 NC_001134.8:g.140631_140632ins*PAD1*). TN10 showed higher muconic acid production compared to TN9 (Additional file 4). We then integrated two copies of the MApw at the *PDC6* locus to create strain TN5 (TN10 *pdc6::*MApw/*pdc6::*MApw). TN5 showed higher production of PCA and muconic acid compared to TN10 (Additional file 5). In these fermentations the level of catechol was always undetectable, which shows that the CDO enzyme confers highly efficient conversion of catechol into muconic acid (Additional files 3, 5).

**Fig. 3:**
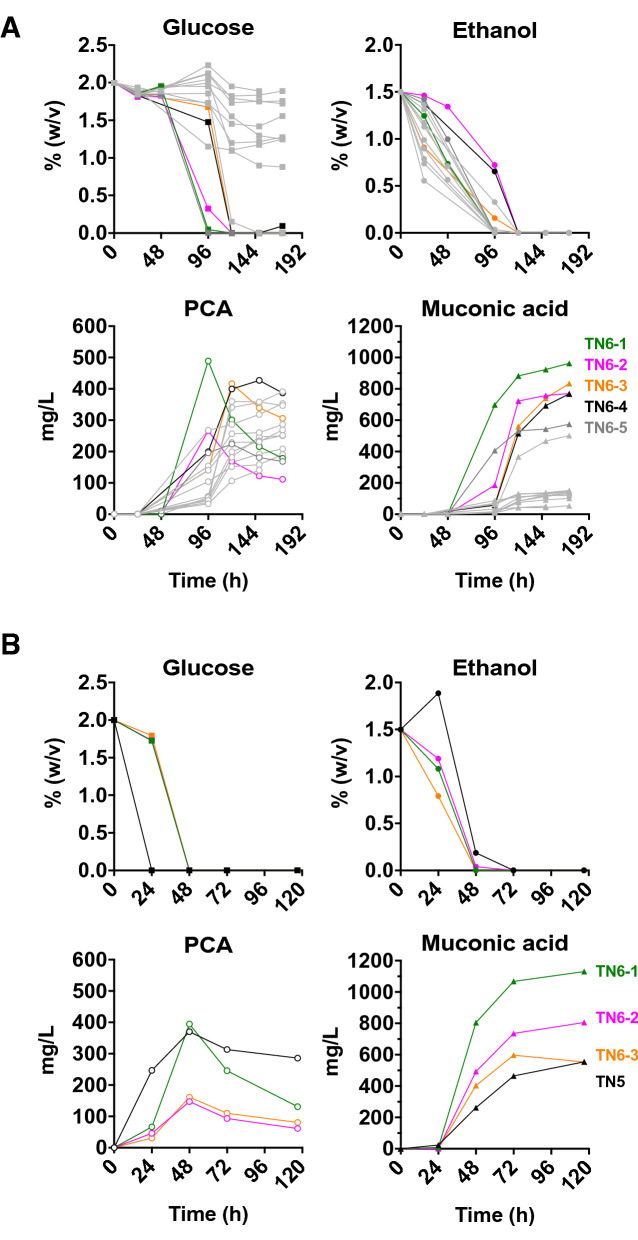
PCA and muconic acid production by the TN5 strain and the TN6 isolates. YP2%D1.6%E medium was used. **A** Performance of all TN6 isolates tested. **B** Comparison of the three best isolates, TN6-1, TN6-2 and TN6-3, with parent strain TN5. Strains were inoculated at OD_600_ 0.1. TN5 (black), TN6-1 (green), TN6-2 (magenta), TN6-3 (orange) and other TN6-X isolates (grey)

Finally, we replaced the two *PDC5* copies with the MApw in TN5 in order to abolish ethanol production completely, resulting in strain TN6 (TN5 *pdc5::*MApw/*pdc5::*MApw). This *pdc-*negative (*pdc−*) strain was selected on solid nutrient medium containing glucose and ethanol as a C2 source to support cytosolic acetyl-CoA synthesis. The TN6 transformants showed large differences in colony size, suggesting the occurrence of additional background mutations improving e.g. glucose tolerance and utilization. Several TN6 transformants with large colony size were evaluated in shake flask cultures with glucose and ethanol as carbon sources for the muconic acid production. In this experiment, the strains were inoculated at a low OD_600_ of 0.1 to allow more extensive growth, which also served as a selection tool to obtain the best transformants (Fig. 3A). The screened isolates showed different rates of glucose utilization, PCA and muconic acid production. They also consumed the ethanol at different rates. One isolate, TN6-1, clearly showed superior behaviour: a rapid glucose utilization and the highest PCA and muconic acid production. Moreover, he TN6-1 isolate showed a strongly transient accumulation of PCA, reaching a maximum titer of 490 mg/L at 96 h and declining to 180 mg/L at 168 h, which correlated to a further increase in muconic acid titer reaching 960 mg/L at 168 h.

The three best performing isolates, TN6-1, TN6-2 and TN6-3, were re-evaluated and compared to the parent strain TN5 in YP medium containing glucose and ethanol. The superior performance of TN6-1 was confirmed, producing 1130 mg/L muconic acid (31.42 mg/g carbon), in contrast to 555 mg/L muconic acid (16.68 mg/g carbon) for TN5 (Fig. 3B). The strains TN6-2 and TN6-3 also produced higher muconic acid concentrations than TN5, but not as pronounced as TN6-1. The residual PCA level was only 130 mg/L for the TN6-1 strain as opposed to 290 mg/L for the TN5 strain (Fig. 3B). All three TN6 isolates showed much slower glucose consumption rates than TN5, reflecting their lower glucose tolerance and utilization despite the presence of the *MTH1* partial deletion and ethanol as C2 source in the medium. However, they all produced muconic acid faster than TN5. On the other hand, TN5 started to produce PCA faster than the TN6 strains and maintained a higher level (Fig. 3B). These results show that some of the TN6 strains with large colony size displayed a much higher flux in the pathway from glucose to muconic acid than the parent strain TN5. The TN5 strain had a low ethanol production (two copies of *PDC5* were still present) and consumed the ethanol after the diauxic shift. The glucose and ethanol consumption rate of the three TN6 isolates was similar, indicating that it cannot be responsible for the superior production of muconic acid by TN6-1.

The five best performing isolates (TN6-1, TN6-2, TN6-3, TN6-4, TN6-5) were whole-genome sequenced to search for possible causative SNPs or other genomic modifications, different from the replacement of *PDC5* by MApw, responsible for their varying performance and in particular for the superior performance of TN6-1. The whole genome sequence of the TN5 strain served as reference. In the different TN6 transformants, we found changes in ploidy for several (parts of) chromosomes (Additional file 6: Table S1) and common (Additional file 7: Table S2) and unique (Additional file 8: Table S3) non-synonymous SNPs in many unrelated genes. Moreover, we observed that several TN6 transformants acquired SNPs in some MApw genes (Additional file 9: Table S4). The I218V SNP in the PCAD enzyme of TN6-1 stood out due to the increased conversion efficiency of PCA to muconic acid in this strain. Hence, the *aroY-C*^*iso, I218V*^ allele was reverted to its wild type form *aroY-C*^*iso*^ in the TN6-1 strain but this did not result in a different production profile, titer or yield for both PCA and muconic acid (Additional file 10). The sequencing data showed that the TN6 transformants displayed a considerable number of other SNPs and (partial) chromosomal ploidy changes that were difficult to disentangle in a straightforward way to investigate their possible involvement in the differences in performance and in particular the superior performance of strain TN6-1.

The use of a low pitching rate for TN6-1 under the obligatory aerobic conditions for muconic acid production led to very active propagation of the yeast resulting in a high loss of carbon into biomass formation instead of muconic acid production. For the next experiments, the pitching rate was therefore increased to OD_600_ 4 (± 1.4 g DW/L). It resulted in higher muconic acid production and also in more complete xylose utilization (Additional file 11). A strong and sudden decrease in extracellular pH was also observed (Additional file 12), which we could partially counteract by the addition of a 50 mM citrate buffer with an initial pH of 5.5 (Additional file 13). This also improved sugar consumption and muconic acid production. Although increasing the citrate buffer concentration to 100 mM helped to increase the buffering capacity even further, it had an inhibitory effect on the fermentation (Additional file 14). We also evaluated higher inocula up to OD_600_ 32 (Additional file 15) with OD_600_ 16 (± 5.4 g DW/L) resulting in the highest production of muconic acid.

**Fig. 4:**
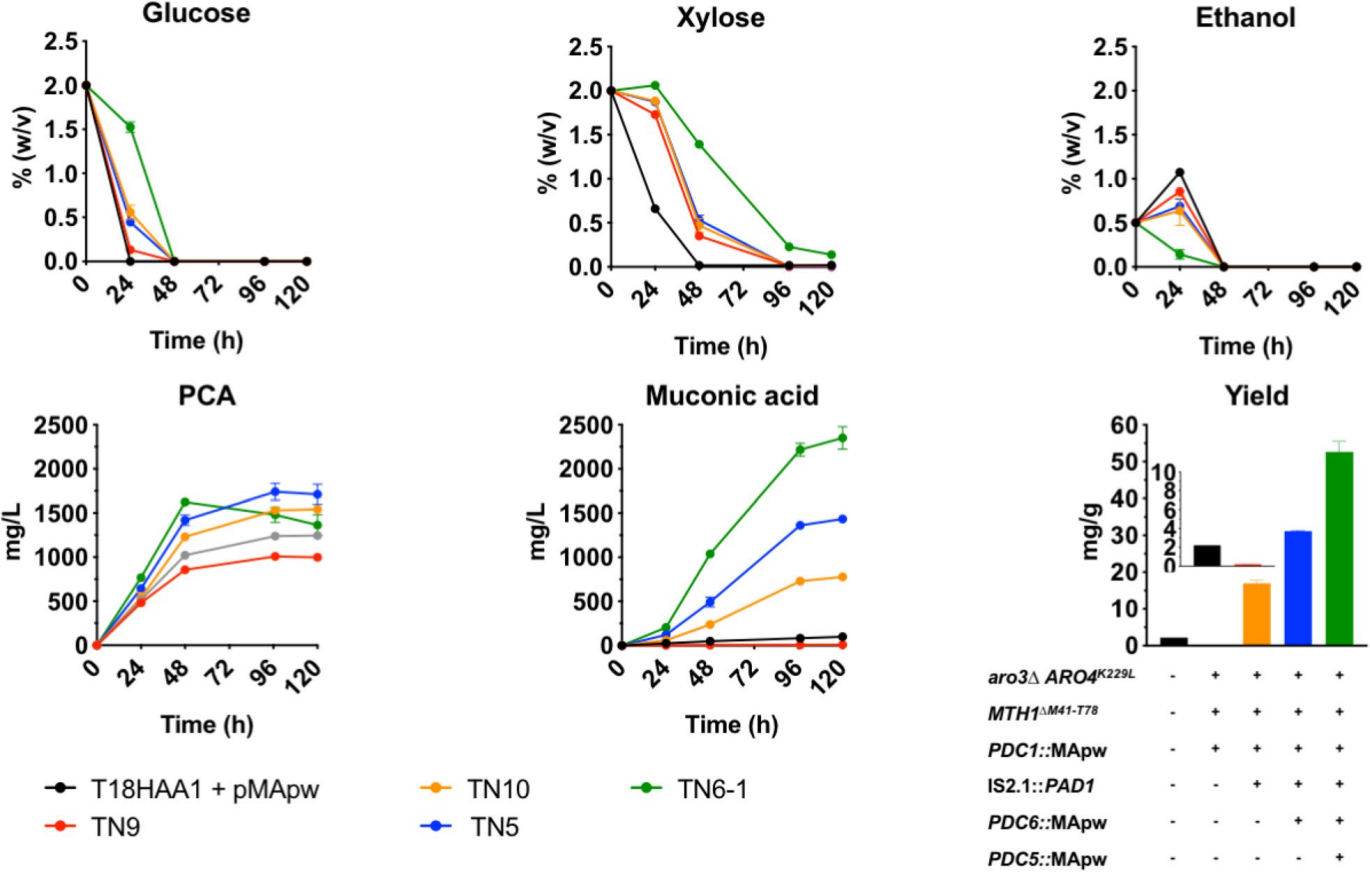
Comparison of muconic acid fermentation performance in the sequentially constructed strains from T18HAA1 up to TN6-1. YP2%D2%X0.5%E medium was used, buffered with 50 mM citrate buffer at an initial pH of 5.5. Strains were inoculated at OD_600_ of 4. Yield represents milligram of muconic acid produced per gram of initial carbon (glucose, xylose and ethanol), by the respective strains with corresponding genomic alterations indicated. Results are means of two biological replicates. Error bars show standard deviation at each time point

Finally, the sequentially developed strains, T18HAA1 + pMApw, TN9, TN10, TN5 and TN6-1, were compared under the same conditions: YP medium buffered with 50 mM citrate buffer at an initial pH of 5.5, containing 2% glucose or 2% xylose supplemented with 0.5% ethanol and an initial pitching of OD_600_ 4 (Fig. 4). There was a clear gradual increase in muconic acid production during strain development with the TN6-1 strain showing clearly superior performance. It reached a titer of 2350 mg/L with a yield of 53 mg/g carbon, in spite of its slower glucose and xylose consumption rate. It was the only strain without additional ethanol production. All strains still produced high levels of PCA, with TN6-1 the only strain showing a transient increase. The increase in the combined amount of PCA and muconic acid produced correlated with the increase in copy number of the genomically integrated MApw.

### Stimulation of PCA conversion to muconic acid in the TN6-1 strain

**Fig. 5:**
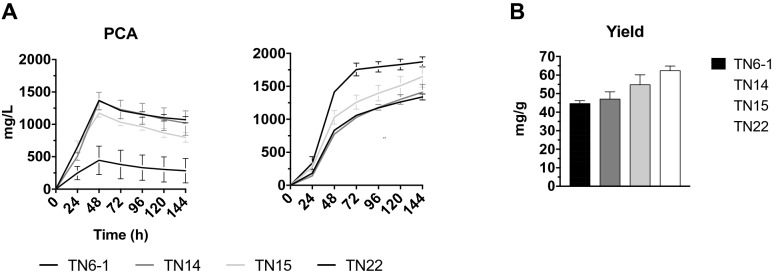
PCA and muconic acid production by the TN6-1, TN14, TN15 and TN22 strains. **A** YP2.5%D0.5%E medium was used buffered with 50 mM citrate buffer at an initial pH of 5.5. Strains were inoculated at OD_600_ of 4. Results are means of two biological replicates for TN6-1 and three independent isolates for the other strains. Error bars show standard deviation at each time point. **B** Yield of muconic acid (mg/g) from the fermentation shown in (**A**) at time point 144 h

Successful approaches to stimulate PCA conversion to muconic acid reported in literature include increasing the copy number of PCAD and related co-factor producing enzyme [25] or expressing co-factor independent PCAD enzymes [20]. We therefore integrated two additional copies of different heterologous genes, encoding PCAD, in the genome of TN6-1: the co-factor independent *AGDC1* from *A. adeninivorans* and *TaGDC1* from *T. atroroseus,* as well as the co-factor dependent *aroY-C*^*iso*^ from *K. pneumoniae*. The constructs were inserted at the integration site IS16.2 of Chr. XVI (NC_001148.4:g.621736) yielding strains TN14, TN15 and TN22, respectively. To avoid possible feedback inhibition by muconic acid on the production rate, we used a low initial glucose concentration of 2.5% and addition of 0.5% ethanol as a C2 source. The TN14 and TN15 strains showed none or a slight improvement in the conversion of PCA, respectively, compared to the TN6-1 strain (Fig. 5A). However, in the TN22 strain, a strong reduction in PCA accumulation with a concomitant increase in muconic acid production was observed. This resulted in an enhanced yield of 62.3 mg/g carbon, compared to 44.6 mg/g carbon for TN6-1 (Fig. 5B).

### Maximal muconic acid production and toxicity of muconic acid on the TN22 strain

**Fig. 6:**
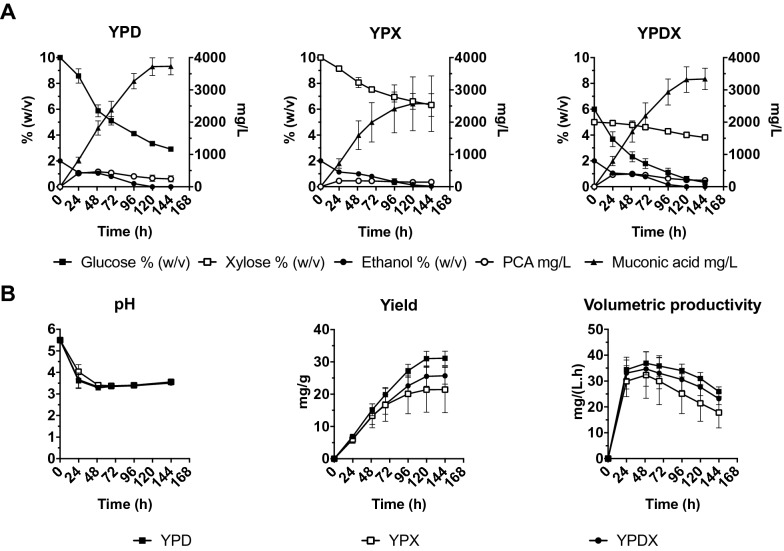
PCA and muconic acid production by the TN22 strain at high pitching rate (OD_600_ 16) and a high initial concentration of either glucose, xylose or a mixture of glucose and xylose. **A** YP medium containing 10% glucose (YPD), 10% xylose (YPX) or 6% glucose and 5% xylose (YPDX) was used, buffered with 50 mM citrate buffer at an initial pH of 5.5. **B** Extracellular pH, muconic acid yield and muconic acid volumetric productivity as a function of time during the fermentations shown in (**A**). Results are means of the three independent replicates. Error bars show standard deviation at each time point

Next, we tested the maximum titer of muconic acid that could be reached by the TN22 strain with glucose and xylose separately, and with a mixture of the two. To reduce the biomass formation we increased the pitching rate to an OD_600_ of 16 (± 5.4 g DW/L), since this was previously found to lead to the highest glucose consumption and muconic acid production (Additional file 15). The TN22 strain was inoculated in shake flasks with YP medium containing 10% glucose (YPD), 10% xylose (YPX) or 6% glucose and 5% xylose (YPDX) with the addition of 2% ethanol for all conditions. The medium was buffered with 50 mM citrate buffer at a starting pH of 5.5. Muconic acid and PCA production were measured over time up to 144 h (Fig. 6). When glucose or xylose were used separately as carbon sources, both were converted into muconic acid, but glucose more efficiently, reaching titers of about 3700 mg/L and 2500 mg/L, respectively. For xylose, the variation between replicates was larger, reaching 3400 mg/L in the best replicate (Fig. 6A). The production rate of muconic acid was higher with glucose compared to xylose, which resulted in a higher muconic acid yield with glucose than with xylose (Fig. 6B). When the medium contained a mixture of glucose and xylose, glucose was consumed preferentially over xylose, with poor consumption of the latter. In all cases, the extracellular pH dropped to 3.3 in 24 h and was maintained at around 3.5. Volumetric productivity was the highest at 48 h with glucose as the substrate (37 mg/L h), followed by the mixture of glucose and xylose (34.7 mg/L h) and then xylose (32.3 mg/L h). Muconic acid titers were higher when glucose was present in the medium. The highest mean muconic acid titer was 3700 mg/L with glucose, then 3300 mg/L with a mixture of glucose and xylose, and 2600 mg/L with xylose.

In all cases, sugar consumption slowed down when muconic acid titers reached around 2000 mg/L, and this was more pronounced for xylose. This suggested feedback inhibition by the increasing muconic acid level in the medium. Hence, we tested muconic acid toxicity using growth assays in media supplemented with various levels of muconic acid. It should be mentioned that the *cis*,*cis* muconic acid, the isomer that is produced during the fermentation, spontaneously isomerizes to cis,trans at pH values lower than 4 [4]. Moreover, when fully protonated, *cis*,*cis* muconic acid has a low solubility of 1 g/L in water, while *cis*,*trans* muconic acid has a solubility of up to 5.2 g/L [53]. The low final pH level, the temperature of fermentation (30 °C) and the high level of muconic acid obtained in our experiments suggested that under our conditions the produced *cis*,*cis* isomer spontaneously isomerized to the *cis*,*trans* isomer.

To test the toxicity, Dr. Ibrahim Khalil (CSCE, KU Leuven) provided a small amount of *cis*,*cis* muconic acid that was isomerized to *cis*,*trans* muconic acid. Different muconic acid concentrations of 0 mg/L, 2000 mg/L and 5000 mg/L were added to YP medium containing 10% glucose, 1% ethanol and 50 mM citrate buffer. The initial pH was set to 4.4 instead of 5.5, to mimic the lower pH level at the end of the fermentation where muconic acid seemed to be inhibitory, while allowing higher solubility of the cis,trans isomer. The TN22 strain was inoculated at an initial OD_600_ 0.1 and OD_600_ 16. In both cases, the presence of 2000 mg/L and 5000 mg/L lowered or severely compromised glucose consumption, muconic acid production and growth of the TN22 strain (Table 3).

**Table 3:** Determination of muconic acid toxicity on strain TN22

Initial OD_600_	MA added (mg/L)	Glucose consumed, % (w/v)	MA produced	Final OD_600_
0.1	0	3.8 (± 0.13)	1660 (± 46)	24.3 (± 1.2)
	2000	3.5 (± 0.16)	870 (± 33)	24.0 (± 1.0)
	5000	0.09 (± 0.004)	29.7 (± 29.7)	7.9 (± 0.1.9)
16	0	6.9 (± 0.08)	3610 (± 16.6)	44.15 (± 0.75)
	2000	4.8 (± 0.08)	1100 (± 47.1)	35.1 (± 0.4)
	5000	2.4 (± 0.1)	0	30.4 (± 0.3)

The TN22 strain was inoculated in YP10%D2%E medium with initial pH 4.4 containing different levels of *cis*,*trans* muconic acid. Two different pitching rates of OD_600_ 0.1 and OD_600_ 16 were used and the fermentations were stopped after 137 h and 168 h, respectively. Glucose consumption, muconic acid production and final OD_600_ were measured. Results show means and standard deviation of two biological replicates.

From the toxicity tests we can conclude that muconic acid is already exhibiting an inhibitory effect on the TN22 strain at titers of approximately 2900 mg/L (2000 mg/L added + 870 mg/L produced). Moreover, the muconic acid production was more sensitive to the presence of elevated muconic acid concentrations in the medium than the growth or glucose consumption of the yeast.

### In-situ extraction of muconic acid during batch fermentations

To address the presumed feedback inhibition of muconic acid on its production, we explored in-situ product removal (ISPR) of the muconic acid during the fermentation. The Bio Base Europe Pilot Plant (Ghent, Belgium) performed a preliminary screening of various solvents and their biocompatibility for extraction of the muconic acid (see “Materials and methods” section). The screening identified polypropylene glycol 4000 (PPG) as an effective and biocompatible solvent for extraction of muconic acid, with a calculated distribution coefficient (Kd) of 5.78.

**Fig. 7:**
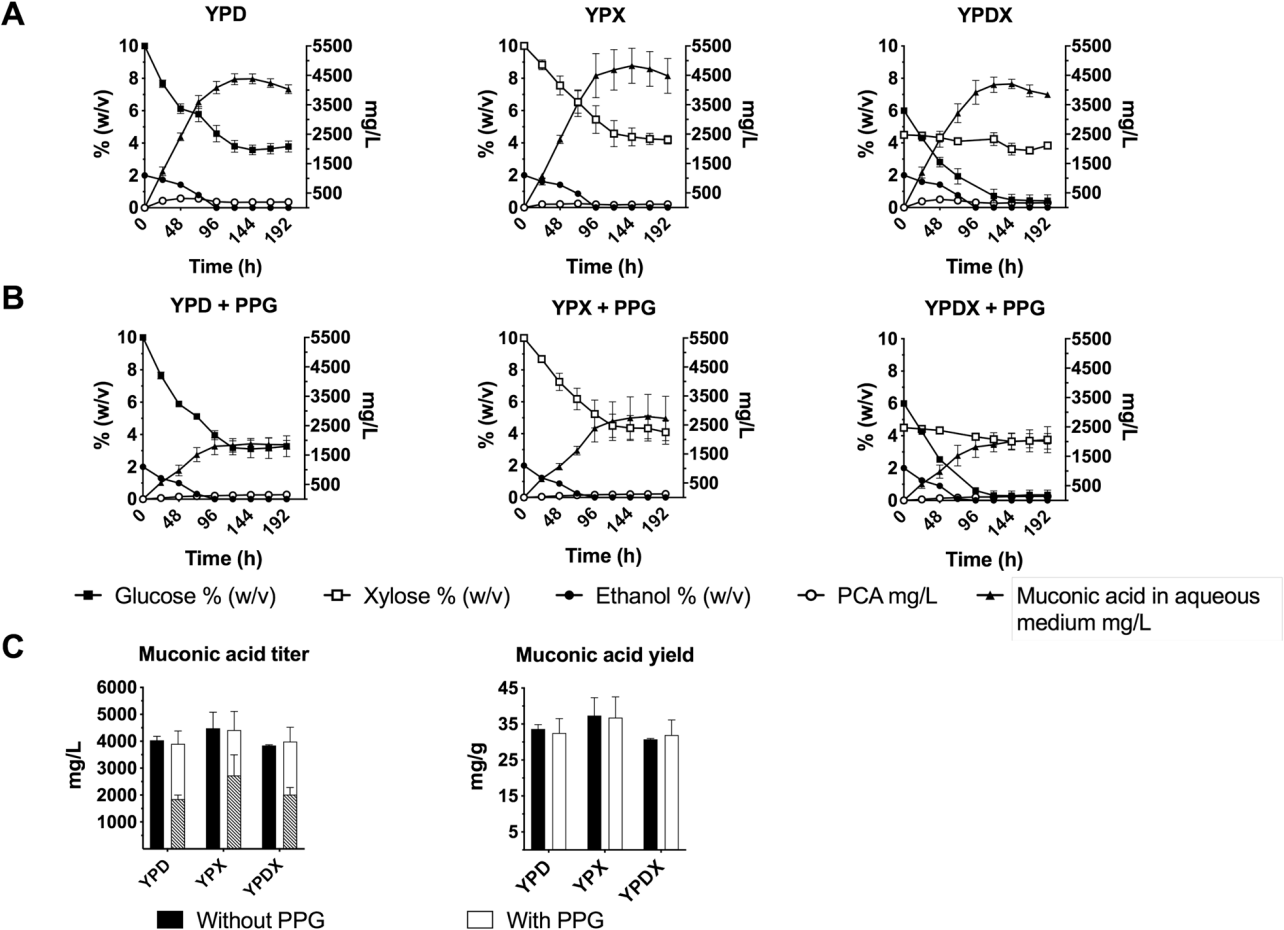
PCA and muconic acid production by the TN22 strain in the absence or presence of the PPG solvent (1:5 ratio). YP medium containing 10% glucose (YPD), 10% xylose (YPX) or 6% glucose and 4.5% xylose (YPDX) with 2% ethanol, buffered with 50 mM citrate buffer at initial pH of 5.5 and inoculum OD_600_ 16. Media contained either **A** no PPG or **B** presence of PPG (solvent/fermentation medium 1:5 ratio). **C** Muconic acid titer and yield (aqueous phase in grey plus solvent phase in white) (at 192 h) in the absence (**A**) and presence (**B**) of PPG. Muconic acid was extracted from the PPG solvent phase using 50% ethanol. Results are means of three independent replicates. Error bars show standard deviation at each time point

We then tested the effect of adding PPG to the fermentation with the TN22 strain. The TN22 strain was inoculated at OD_600_ 16 in shake flasks with YP medium containing 10% glucose, 10% xylose or a mixture of the two (6% glucose, 4.5% xylose), and 2% ethanol as a C2 source, buffered with 50 mM citrate buffer at an initial pH of 5.5. The PPG was added at the beginning of the fermentation in a 1:5 ratio to the medium volume (i.e. 40 mL YP medium and 8 mL PPG). Under these conditions, the amount of muconic acid that accumulated in the fermentation medium was reduced by about 50% (Fig. 7B) compared to the control (Fig. 7A). To determine the amount of muconic acid in the PPG layer, we separated the layer from the aqueous phase and washed it with 4 mL 50% ethanol [54], and measured the muconic acid concentration in the ethanol using HPLC analysis. This showed that almost equal amounts of muconic acid could be recovered from the PPG phase compared to the amount present in the fermentation medium (Fig. 7C). In all cases, the titer and yield of the total amount of muconic acid produced were similar to those in the fermentation without PPG (Fig. 7C). In spite of the strong reduction by about 50% of the muconic acid titer in the fermentation medium in the presence of PPG, the sugar consumption still arrested after approximately half of the initial sugar concentration was utilized (Fig. 7B), similar to the fermentation in the absence of PPG (Fig. 7A). These results contradict that product inhibition by muconic acid is responsible or at least solely responsible for the arrest of sugar utilization. They suggest that another (possibly metabolic) bottleneck is building up during the production of muconic acid, which inhibits and finally stops the sugar consumption and muconic acid production.

The slow-down and subsequent arrest of sugar consumption cannot be due to a loss of the yeast’s viability since we did not observe a pronounced drop in the number of colony forming units (CFU) up to 144 h in the fermentation with any of the sugar substrates. Only after 192 h, and only in the fermentation with glucose, a strong drop in CFU was noticed, which was not affected by the presence of PPG (Additional file 16).

To confirm these conclusions, we repeated the muconic acid fermentations under the same conditions but with addition of PPG at the beginning of the fermentation in a 1:2 ratio to the medium volume (i.e. 40 mL YP medium and 20 mL PPG). This resulted in a further reduction of the muconic acid concentration in the medium and a concomitant increase in the PPG phase, but without any significant change in the sugar utilization pattern and total muconic acid production compared to the fermentations with a PPG to medium ratio of 1:5 (Additional file 17). The same observations were also made for the viability measurements. Only in the muconic acid production with glucose, there was a strong drop in CFUs late in the fermentation, which was not overcome by the presence of PPG. On the other hand, in the fermentations with xylose and mixed glucose and xylose, there was only a moderate drop in CFUs, which was also not affected by the presence of PPG (Additional file 18).

## Discussion

The goal of the present work was to construct a second-generation yeast cell factory with efficient production of muconic acid from both glucose and xylose. We utilized the xylose-utilizing and inhibitor tolerant GSE16-T18HAA1 as a starting strain [46]. Several known strategies are crucial in tailoring the central metabolism of *S. cerevisiae* to achieve high titers of muconic acid [19–21, 55]. These all focus on increasing the DHS substrate availability by improving flux towards (*TKL1* overexpression) and in the shikimate pathway (expression of *ARO4*^*K229L*^ and *ARO3*^*K222L*^) or lowering DHS breakdown towards aromatic amino acids (*ARO1*^*aroEΔ*^). The starting strain GSE16-T18HAA1 used in this work overexpressed all the non-oxidative pentose phosphate pathway genes, including *TKL1* [12], which was reported to boost E4P availability. The flux towards the shikimate pathway in this strain was further increased by deleting *ARO3* and expressing the feedback resistant *ARO4*^*K229L*^. Overexpression of *ARO4*^*K229L*^ and *ARO3*^*K222L*^ has also been reported in the literature [20], but we were not able to obtain stable strains with these modifications. The choice for the heterologous genes used in this work is largely based on previous studies (Table 4), with exception of PCAD. We opted for the *aroY-C*^*iso*^ isoform due to its reduced inhibition by oxygen [17]. Improvement of PCA conversion has been achieved by increasing the copy number of the gene encoding PCAD and its co-factor availability [29, 39, 56]. Despite the fact that the TN6 strain already expressed 6 copies of the gene encoding PCAD (2 copies in each of the *PDC* loci) and had enhanced prFMN production due to insertion of two *PAD1* overexpression cassettes, PCA still accumulated to high levels (Fig. 4). To address this problem we explored the additional expression of the prFMN-dependent enzymes *aroY-C*^*iso*^ and the prFMN-independent PCAD enzymes (*aGDC1* and *TaGDC1*) [20]. As opposed to the results in this previous work, we only observed significant improvement of PCA conversion resulting in higher muconic acid levels when we expressed two additional copies of the *aroY-C*^*iso*^ (Fig. 5). Thus, the level of prFMN does not seem to be a limiting factor for efficient muconic acid production in our strain background.

**Table 4:** Comparison of titer and yield for reported *S. cerevisiae* muconic acid cell factories

Strain name	Genetic characteristics	Fermentation type (total h)	Carbon source	Titer (mg/L)	Yield (mg/g)	Refs
CEN.PK2-1C^*aroED*^	*ARO1*^*aroE*^^*Δ*^*, aroZ*_*Bth*_*, asbF*_*Bth*_*, aroY-C*^*iso*^	Batch (170 h)	D	1.56	0.78	[17]
MuA12	*aro3Δ, zwf1Δ, ARO4*^*K229L*^*, TKL1 aroZ*_*Pa*_*, aroY*_*Ecl*_*, HQD2*_*Ca*_	Batch (108 h)	D	141	3.53	[21]
MA4	*ric1Δ, aro1Δ, ARO4*^*K229L*^*, ARO1*^*D14091,D920A*^*, TKL1, aroZ*_*Kp*_*, aroY*_*Kp*_*, HQD2*_*Ca*_	Batch (72 h)	D	2.7	33.57	[23]
MuA-5.01.1.02	*TKL1, ARO1*^*aroE*^^*Δ*^*,aroZ*_*Pa*_*, aroY*_*Ecl*_*, HQD2*_*Ca*_	Fed-batch (240 h)	D	2.1	12.9	[24]
JTY4	*zwf1Δ, TKL1, ARO3*^*K222L*^*, ARO4*^*K229L*^*,ARO1*^*aroE*^^*Δ*^* ARO1*^*K1370A*^*, aroZ*_*Pa*_*, TaGDC1, catA*_*Ar*_	Batch (120 h)	D	1244	31	[20]
Isolate 7	*TKL1, aroZ*_*Pa*_*, aroY-B, aroY-C*^*iso*^*, aroY-D, catA*_*Ca*_	Fed-batch (120 h)	D	2008	13.4	[25]
Aro1_PEST_-Hqd2(p6G)	*aro3Δ, aro4Δ, ARO1-CLN2*_*PEST*_*, PAD1, ARO4*^*K229L*^*, aroB*_*Ec*_*, aroD*_*Ec*_*, aroZ*_*Pa*_*, aroY*_*-*_*C*^*iso*^*, HQD2*_*Ca*_	Fed-batch (168 h)	D	5.1	58	[22]
ST8943	*ARO4 *^*K229L*^*, ARO1*^*aroED*^*, aroZ*_*Pa*_*, aroY-C*^*iso*^*, aroY-B, aroY-D, catA*_*Ca*_	Fed-batch (150 h)	D	20,800	66.2	[39]
Ic0.09-31	XK*,* PPP*, ARO1*^*aroED*^*, XylA*_*Bv*_* aroZ*_*Nc*_*, aroY*_*Ci*_*,CatA*_*Cn*_	Batch (72 h)	DX	424	3.26	[14]
TN22	*aro3Δ, ARO4*^*K229L*^*, PAD1,TKL1, aroZ*_*Pa*_*,, aroY-C*^*iso*^*, HQD2*_*Ca*_	Batch (192 h)	^a^D/X/DX	4030/4500/3840	33.6/37.3/30.7	This study

*D* glucose, *X* xylose

^a^Ethanol was added to the fermentation medium to address C2 auxotrophy of the *pdc-*strain. Fermentations were performed in rich YP medium, buffered with citrate buffer at pH 5.5 with an initial OD_600_ 16

By applying these approaches and also eliminating ethanol production while increasing the copy number of the MApw, we were able to obtain a maximum titer of 4.5 g/L muconic acid in batch fermentations with glucose and xylose. This is the highest titer reported in batch fermentations up to now (Table 4) and also the first report of muconic acid production without concomitant ethanol production.

The yeast *S. cerevisiae* has a very high capacity and preference for ethanol production [32]. No previous studies have focussed on completely eliminating ethanol production by deleting the *PDC* genes for improving muconic acid production. Instead, they focussed on other approaches, such as lowering ethanol production by optimizing the feed composition and rate in fed-batch fermentations [39]. To eliminate ethanol production, we deleted the *PDC1*, *5* and *6* genes [34, 37, 57] and simultaneously integrated the MApw genes in these loci. Because of this approach it is difficult to determine to what extent the absence of ethanol production and/or the increased copy number of the MApw was responsible for the observed increase in muconic acid production.

Upon deleting the final *PDC* gene, *PDC5*, we obtained multiple transformants with highly varying performance. Of these transformants, TN6-1 showed the highest muconic acid production and the best conversion of PCA to muconic acid (Fig. 3). The differences in performance were likely caused by background mutations occurring in the transformants. In addition, pyruvate is known to accumulate in pdc-strains causing cytosolic acidification and a metabolic imbalance [32, 36]. Hence, the unknown mutations present in the TN6-1, TN6-2 and TN6-3 strains may lower the pyruvate accumulation for instance by increasing the conversion of pyruvate via PEP and E4P into the shikimate pathway causing higher muconic acid production. Because of the multiple genetic modifications present, we were so far unable to pinpoint the causative mutation(s) in these transformants (Additional files 7, 8, 9 and 10). Further detailed investigation, however, may likely reveal novel promising approaches to improve muconic acid production.

When we used higher initial sugar concentrations in order to obtain higher muconic acid titers, we observed stuck fermentations both with glucose and xylose, and a mixture of the two. The accumulation of about 3–4 g/L muconic acid always seemed to limit further production, irrespective of the level of PCA (Figs. 6A and 7A). Since we also found muconic acid to be toxic and strongly inhibiting at low pH its own production already at levels around 2 g/L (Table 3), we envisaged the stuck muconic acid fermentations to be due to muconic acid toxicity. Previous work also reported muconic acid toxicity to be a limiting factor, especially at lower extracellular pH levels near the p*Ka* (3.64) of muconic acid [39]. In that report, toxicity of the increasing concentrations of muconic acid was prevented by maintaining the extracellular pH at 6 in controlled fed-batch fermentations. This resulted in the more tolerable deprotonated form of muconic acid being the dominant form and allowed to reach a maximum titer of 20.8 g/L. An alternative or additional explanation is that the fed-batch process kept the sugar level continuously low so that the unknown bottleneck for muconic production that apparently built up in our strain during batch fermentation with high initial sugar levels, was never generated. In another study, Suastegui et al. also used high initial glucose concentrations of 8%, which resulted in a muconic acid titer of 2.7 g/L and an incomplete consumption of glucose with 2.5% left after 72 h in batch fermentation [23]. This fits with our observations that high initial sugar levels result in incomplete muconic acid fermentations.

Hence, we sought to further improve the muconic acid production by applying an ISPR approach using the PPG solvent, which could be essential for establishing a promising process for industrial application with the appropriate specifications for titer, productivity and yield. The addition of the PPG solvent resulted indeed in the efficient removal of approximately half (Fig. 7) or more than half (Additional file 17), depending on the PPG/medium volume ratio, of the muconic acid from the fermentation medium. However, in spite of the strong reduction in the muconic acid concentration in the fermentation medium, there was no improvement in sugar utilization and the fermentations remained stuck as in the absence of PPG. CFU measurements showed that this was not due to a loss in viability (Additional files 16, 18). The addition of PPG in a 1:2 ratio reduced the muconic acid concentration to levels between 500 and 1500 mg/L (Additional file 17). Such levels did not cause any inhibition of sugar utilization when lower initial sugar levels were used (Fig. 4, Additional files 10, 11, 12, 13 and 14). Hence, we are bound to conclude that during the muconic acid fermentation with high initial sugar levels an unidentified problem is generated, which starts to inhibit and finally completely blocks sugar consumption and muconic acid production. This could be a bottleneck in metabolism, such as a build-up of a metabolic intermediate, that starts to become toxic at high concentrations. Further research is needed to identify this specific problem and also whether it occurs in all strains or is related to the genetic modification introduced or to a mutation spontaneously generated in the TN22 strain. On the other hand, the similar result of incomplete sugar utilization reported by Suastegui et al. when using a high initial sugar concentration, suggest that it is independent of the genetic background and also of the pdc-genotype [23].

Although our approaches were successful in achieving a high titer of muconic acid both with glucose and xylose, there is still need for further substantial improvement. The following points can be addressed in future research.

First, detailed metabolomic analysis is required to identify the cause of the stuck muconic acid fermentations when high initial sugar levels are used. The latter are required to obtain high muconic acid titers. When the cause can be identified appropriate genetic modifications have to be introduced to overcome the problem. Alternatively, or when the problem cannot be identified in a straightforward manner, evolutionary adaptation could be explored to generate strains able to continue growth and sugar utilization over a longer time and thus outcompete the current strains that arrest growth and sugar utilization rapidly.

Second, although we had engineered an internal deletion in *MTH1,* it only partially overcame the C2 requirement and ethanol addition was still necessary for efficient sugar consumption. Other approaches to address the C2 requirement have been proposed, but none of these fully overcame the C2 problem [33, 58]. Evolutionary adaptation of the TN22 strain to obtain C2 independency might be more effective. At the same time the evolutionary adaptation might also lead to a higher muconic acid productivity.

Third, it is clear that muconic acid is toxic to *S. cerevisiae*, even at relatively low concentrations of 4–5 g/L especially at low pH. This is important for muconic acid production with glucose, but even more with xylose, since xylose utilization is more sensitive to stress factors, like the presence of organic acids especially at low pH [59]. The xylose-utilization capacity of the TN22 strain was also reduced in the presence of glucose. This is a general problem with second-generation yeast strains [60]. To reach the high muconic acid titer, yield and productivity required for industrial implementation, ISPR of the muconic acid will be required. Our work has shown that PPG is a very effective solvent to remove muconic acid from the fermentation medium. Once the cause of the stuck fermentations has been addressed, application of ISPR with PPG may be highly effective in further increasing the maximal muconic acid titer obtained.

Fourth, the obligatory aerobic conditions result in significant biomass formation and loss of yield. Different approaches can be explored to reduce the growth capacity of the yeast under muconic acid production conditions.

## Conclusion

We successfully constructed a second-generation yeast cell factory for the production of muconic acid with glucose and xylose as substrates. Ethanol production was completely eliminated and also production of the PCA intermediate was minimized. A maximal muconic acid titer of 4.5 g/L was achieved, the highest obtained in batch fermentations with yeast up to now. We demonstrated toxicity of muconic acid in the concentration accumulated, for growth, sugar utilization and especially muconic acid production itself. High initial sugar levels resulted in stuck fermentations. Although ISPR with PPG reduced the muconic acid concentration in the medium to less than half, the arrest of sugar utilization and final drop in viability were not affected. This appears to indicate that an unknown, possibly metabolic bottleneck builds up during muconic acid fermentation with high sugar levels, which inhibits and finally terminates all further sugar utilization and muconic acid production.

## Supplementary Information

**Additional file 1.** PCA and muconic acid production by the TN7 strain expressing the MApw plasmid. Media containing (A) glucose, (B) xylose or (C) a mixture of glucose and xylose as substrate. Strains were inoculated at OD_600_ 1. Results are the means of three independent replicates for each time point. Error bars show standard deviation at each time point.

**Additional file 2.** PCA and muconic acid production by the TN9 strain expressing two copies of the MApw at the *PDC1 *locus. Media containing (A) glucose, (B) xylose or (C) a mixture of glucose and xylose as carbon source. Strains were inoculated at OD_600_ 1. Results are the means of three independent replicates for each time point. Error bars show standard deviation at each time point.

**Additional file 3.** PCA, catechol and muconic acid production by the TN8 *pdc1∆* strain expressing pMApw and either *PAD1* or *aroY-B* on multi-copy plasmids. Strains were inoculated in YP4%D medium and at OD_600_ 1. Samples were taken after 48 h. Inset shows in more detail the results for muconic acid.

**Additional file 4.** Production of PCA and muconic acid by the TN10 strain, which contains 2 copies of the MApw integrated in the PDC1 locus and 2 copies of the PAD1 overexpression cassette. Media containing (A) glucose, (B) xylose or (C) a mixture of glucose and xylose as carbon source. Strains were inoculated at OD_600_ 1. Results are the means of three independent replicates for each time point. Error bars show standard deviation at each time point.

**Additional file 5.** Production of PCA and muconic acid by the TN10 and TN5 strain, which contains two or four copies of the MApw, respectively. Strains were inoculated at OD_600_ 1 and sampled after 48h (A) and for TN5 also periodically during three days (B). YP containing 2% glucose and 2% xylose was used. Results are the means of two biological replicates for TN10 or three independent replicates for TN5. Error bars show standard deviation at each time point.

**Additional file 6: Table S1.** Ploidy of all chromosomes in the TN5 strain and the different TN6 transformants.

**Additional file 7: Table S2.** Common SNPs in the TN6 transformants compared to TN5.

**Additional file 8: Table S3.** Unique SNPs in each TN6 transformant compared to TN5.

**Additional file 9: Table S4.** SNPs in MApw genes in the TN6 transformants compared to TN5.

**Additional file 10.** PCA and muconic acid production by TN6-1 and TN16. (A) YP2.5%D0.5%E medium buffered with 50 mM citrate buffer and initial pH of 5.5. Strains were inoculated at OD_600_ 4. Results are the means of two biological replicates for TN6-1 and three independent replicates for TN16. Error bars show standard deviation at each time point. (B) Total molar yield of PCA and muconic acid from fermentation experiment shown in (A) at time point 144 h.

**Additional file 11.** Comparison of different pitching rates with TN6-1. YP2%D2.25%X1.6%E medium. Results are means of two biological replicates. Error bars show standard deviation at each time point.

**Additional file 12.** Comparison of different glucose concentrations for the production of PCA and muconic acid by TN6-1. YP medium with 2%, 5% or 10% glucose and 1.6% ethanol. Strains were inoculated at OD_600_ 4. Results are means of two biological replicates. Error bars show standard deviation at each time point.

**Additional file 13.** Comparison of different glucose concentrations for the production of PCA and muconic acid with the TN6-1 strain in YP medium buffered with 50 mM citrate buffer at initial pH of 5.5. YP medium with 2%, 5% or 10% glucose and 1.6% ethanol. Strains were inoculated at OD_600_ 4. Results are means of two biological replicates. Error bars show standard deviation at each time point.

**Additional file 14.** Comparison of different glucose concentrations for the production of PCA and muconic acid by TN6-1 in YP medium buffered with 100 mM citrate buffer at initial pH of 5.5. YP medium with 2%, 5% or 10% glucose and 1.6% ethanol. Strains were inoculated at OD_600_ 4. Results are means of two biological replicates. Error bars show standard deviation at each time point.

**Additional file 15.** Comparison of different pitching rates for the production of PCA and muconic acid with the TN6-1 strain in YP medium buffered with 50 mM citrate buffer at initial pH of 5.5. YP medium with 12% glucose and 1.6% ethanol. Results are means of two biological replicates. Error bars show standard deviation at each time point.

**Additional file 16.** Determination of viability during muconic acid fermentations in YPD, YPX and YPDX. CFUs were determined at different time points during the fermentations with the TN22 strain in the absence or presence of the PPG solvent (1:5 ratio) (Fig. 7) up to 144 h. They are shown on a logarithmic scale. (At 192 h very low and highly variable CFUs were found indicating strong loss of viability after extended incubation at the end of the fermentation.)

**Additional file 17.** PCA and muconic acid production by the TN22 strain in the presence of the PPG solvent (1:2 ratio). YP medium containing 10% glucose (YPD), 10% xylose (YPX) or 5% glucose and 5% xylose (YPDX) with 2% ethanol, buffered with 50 mM citrate buffer at initial pH of 5.5 and inoculum OD_600_ 16. Media contained either (A) no PPG or (B) presence of PPG (solvent/fermentation medium 1:5 ratio). (C) Muconic acid titer and yield (aqueous phase in grey plus solvent phase in white) (at 216 h) in the absence (A) and presence (B) of PPG. Muconic acid was extracted from the PPG solvent phase using 50% ethanol. Results are means of three independent replicates. Error bars show standard deviation at each time point.

**Additional file 18.** Determination of viability during muconic acid fermentations in YPD, YPX and YPDX. CFUs were determined at different time points during the fermentations with the TN22 strain in the absence or presence of the PPG solvent (1:2 ratio) (Additional file 17) up to 216 h. They are shown on a logarithmic scale.

## Data Availability

All data have been stored on dedicated computers at KU Leuven. All data and yeast strains are freely available upon request. The sequencing data have been deposited with links to the BioProject Accession number PRJNA699459 in the NCBI BioProject database (https://www.ncbi.nlm.nih.giv/bioproject/).

## Abbreviations

YPD: Yeast extract peptone dextrose
PCA: Protocatechuic acid
MApw: Muconic acid biosynthesis pathway
DHS: 3-Dehydroshikimate
DHSD: DHS dehydratase
PCA: Protocatechuic acid
PCAD: PCA decarboxylase
CDO: Catechol 1,2-dioxygenase
PDC: Pyruvate decarboxylase
PEP: Phosphoenolpyruvate
E4P: Erythrose-4-phosphate
prFMN: Prenylated flavine mononucleotide
MTY: Maximum theoretical yield
CFU: Colony forming units
PPG: Polypropylene glycol 4000
