# Supplementary material for: In-situ muconic acid extraction reveals sugar consumption bottleneck in a xylose-utilizing Saccharomyces cerevisiae strain
Source: Microb Cell Fact. 2021 Jun 7;20:114. doi: 10.1186/s12934-021-01594-3 (PMC8182918; doi:10.1186/s12934-021-01594-3)
Supplement: Supplementary file 2 — Additional file 2. PCA and muconic acid production by the TN9 strain expressing two copies of the MApw at the PDC1 locus. Media containing (A) glucose, (B) xylose or (C) a mixture of glucose and xylose as carbon source. Strains were inoculated at OD600 1. Results are the means of three independent replicates for each time point. Error bars show standard deviation at each time point. [file 12934_2021_1594_MOESM2_ESM.docx]

**Additional file 2**

**
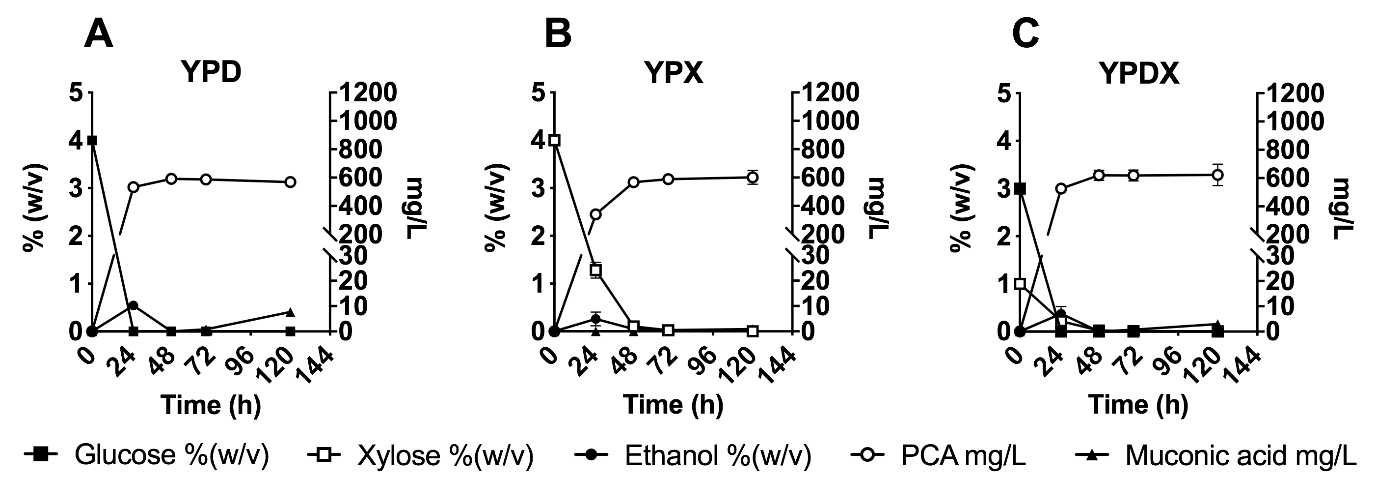
**

**PCA and muconic acid production by the TN9 strain expressing two copies of the MApw at the *PDC1* locus.** Media containing (**A**) glucose, (**B**) xylose or (**C**) a mixture of glucose and xylose as carbon source. Strains were inoculated at OD_600_ 1. Results are the means of three independent replicates for each time point. Error bars show standard deviation at each time point.
