# Supplementary material for: In-situ muconic acid extraction reveals sugar consumption bottleneck in a xylose-utilizing Saccharomyces cerevisiae strain
Source: Microb Cell Fact. 2021 Jun 7;20:114. doi: 10.1186/s12934-021-01594-3 (PMC8182918; doi:10.1186/s12934-021-01594-3)
Supplement: Supplementary file 3 — Additional file 3. PCA, catechol and muconic acid production by the TN8 pdc1∆ strain expressing pMApw and either PAD1 or aroY-B on multi-copy plasmids. Strains were inoculated in YP4%D medium and at OD600 1. Samples were taken after 48 h. Inset shows in more detail the results for muconic acid. [file 12934_2021_1594_MOESM3_ESM.docx]

**
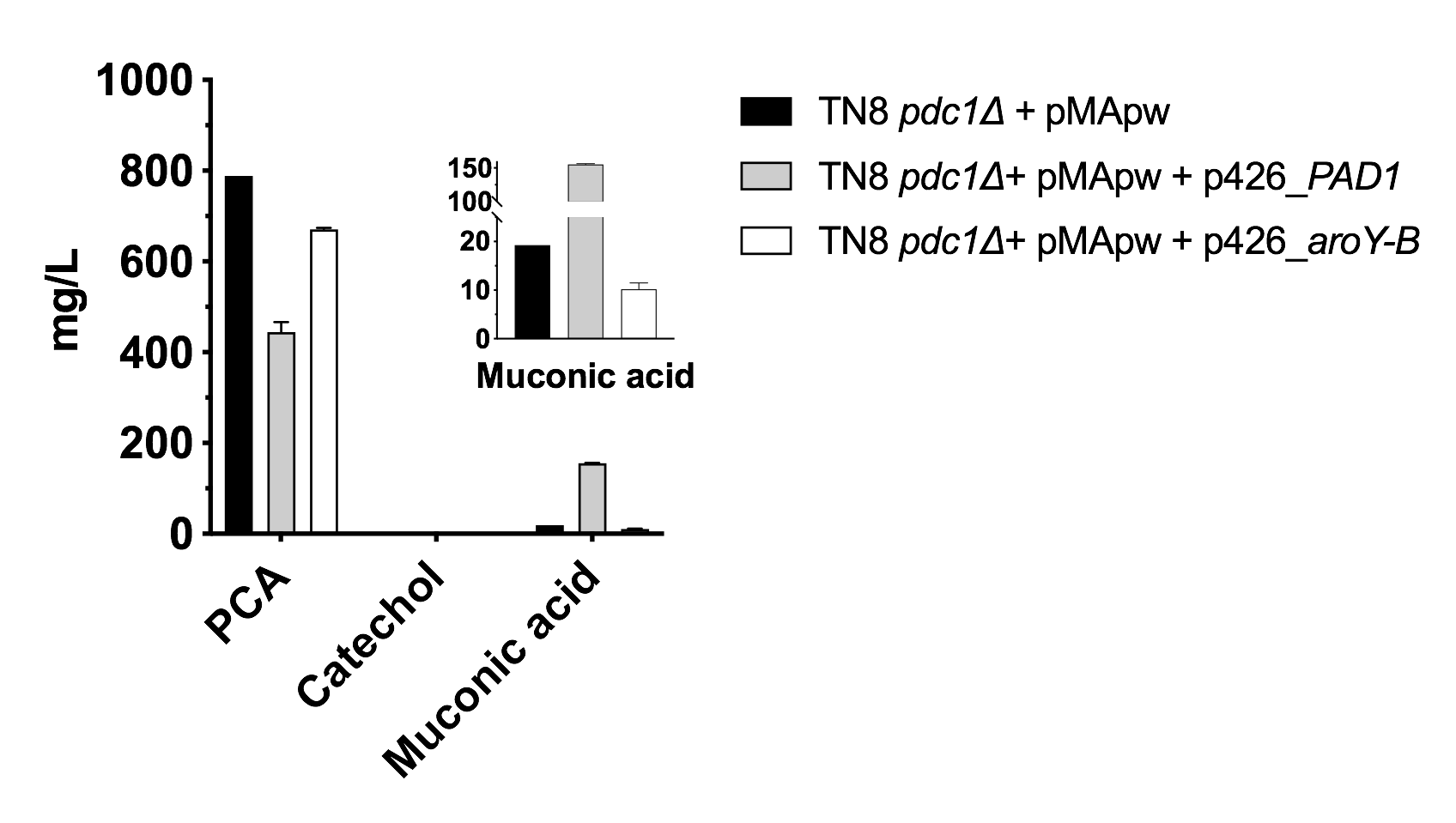
Additional file 3**

**PCA, catechol and muconic acid production by the TN8 *pdc1∆* strain expressing pMApw and either *PAD1* or *aroY-B* on multi-copy plasmids.** Strains were inoculated in YP4%D medium and at OD_600_ 1. Samples were taken after 48h. Inset shows in more detail the results for muconic acid.
