# Supplementary material for: In-situ muconic acid extraction reveals sugar consumption bottleneck in a xylose-utilizing Saccharomyces cerevisiae strain
Source: Microb Cell Fact. 2021 Jun 7;20:114. doi: 10.1186/s12934-021-01594-3 (PMC8182918; doi:10.1186/s12934-021-01594-3)
Supplement: Supplementary file 4 — Additional file 4. Production of PCA and muconic acid by the TN10 strain, which contains 2 copies of the MApw integrated in the PDC1 locus and 2 copies of the PAD1 overexpression cassette. Media containing (A) glucose, (B) xylose or (C) a mixture of glucose and xylose as carbon source. Strains were inoculated at OD600 1. Results are the means of three independent replicates for each time point. Error bars show standard deviation at each time point. [file 12934_2021_1594_MOESM4_ESM.docx]

**Additional file 4**

**
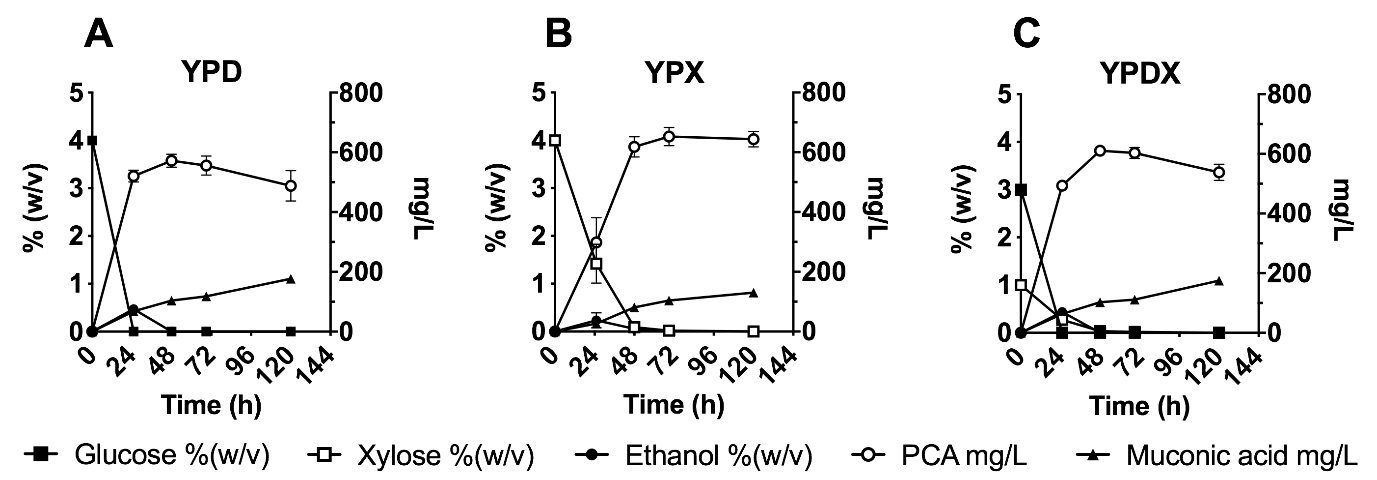
**

**Production of PCA and muconic acid by the TN10 strain, which contains 2 copies of the MApw integrated in the *PDC1* locus and 2 copies of the PAD1 overexpression cassette.** Media containing (**A**) glucose, (**B**) xylose or (**C**) a mixture of glucose and xylose as carbon source. Strains were inoculated at OD_600_ 1. Results are the means of three independent replicates for each time point. Error bars show standard deviation at each time point.
