# Supplementary material for: In-situ muconic acid extraction reveals sugar consumption bottleneck in a xylose-utilizing Saccharomyces cerevisiae strain
Source: Microb Cell Fact. 2021 Jun 7;20:114. doi: 10.1186/s12934-021-01594-3 (PMC8182918; doi:10.1186/s12934-021-01594-3)
Supplement: Supplementary file 5 — Additional file 5. Production of PCA and muconic acid by the TN10 and TN5 strain, which contains two or four copies of the MApw, respectively. Strains were inoculated at OD600 1 and sampled after 48h (A) and for TN5 also periodically during three days (B). YP containing 2% glucose and 2% xylose was used. Results are the means of two biological replicates for TN10 or three independent replicates for TN5. Error bars show standard deviation at each time point. [file 12934_2021_1594_MOESM5_ESM.docx]

**Additional file 5**

**
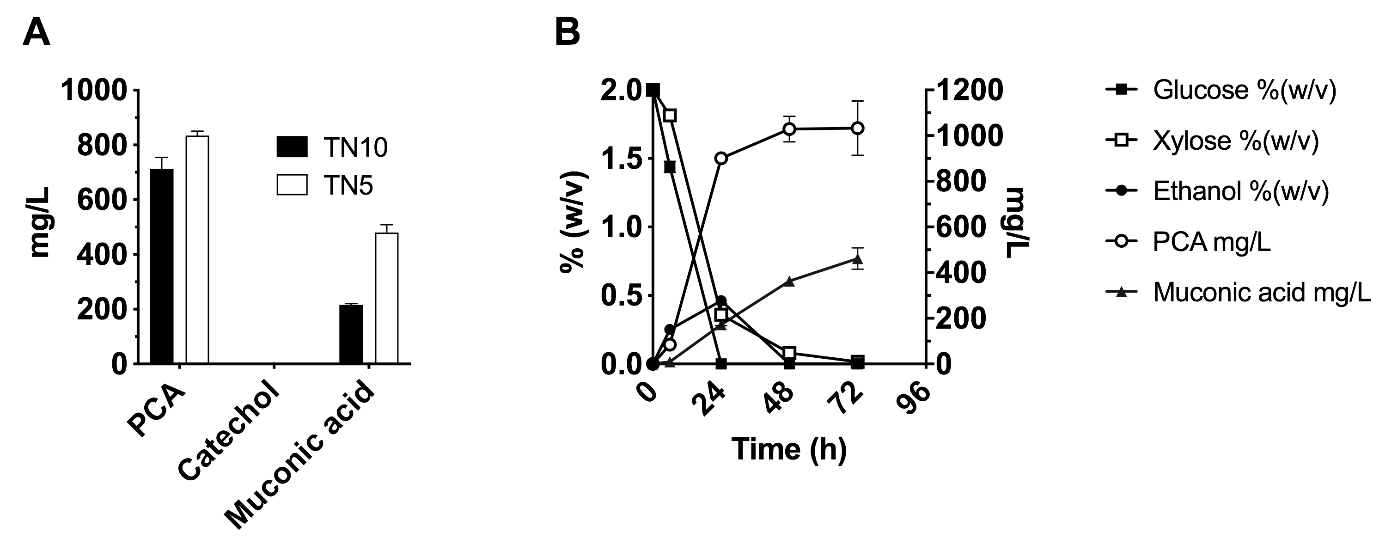
**

**Production of PCA and muconic acid by the TN10 and TN5 strain, which contains two or four copies of the MApw, respectively.** Strains were inoculated at OD_600_ 1 and sampled after 48h (**A**) and for TN5 also periodically during three days (**B**). YP containing 2% glucose and 2% xylose was used. Results are the means of two biological replicates for TN10 or three independent replicates for TN5. Error bars show standard deviation at each time point.
