# Supplementary material for: In-situ muconic acid extraction reveals sugar consumption bottleneck in a xylose-utilizing Saccharomyces cerevisiae strain
Source: Microb Cell Fact. 2021 Jun 7;20:114. doi: 10.1186/s12934-021-01594-3 (PMC8182918; doi:10.1186/s12934-021-01594-3)
Supplement: Supplementary file 6 — Additional file 6: Table S1. Ploidy of all chromosomes in the TN5 strain and the different TN6 transformants. [file 12934_2021_1594_MOESM6_ESM.docx]

**Additional file 6**

**Ploidy of all chromosomes in the TN5 strain and the different TN6 transformants.**

| **Chr.** | **TN5** | **TN6-1** | **TN6-2** | **TN6-3** | **TN6-4** | **TN6-5** |
| --- | --- | --- | --- | --- | --- | --- |
| I | 3n | 3n | 6n | 6n | 3n | 6n |
| II | 2n | 2n | 2n | 2n | 2n | 2n |
| III | 2n | 2n | 4n | 2n | 2n | 4n |
| IV | 2n | 2n | 2n | 2n | 2n | 2n |
| V | 2n | 1n + large part 2n | 2n | 2n | 2n | 2n |
| VI | 2n | 2n | 4n | 4n | 2n | 4n |
| VII | 2n | 2n | 2n | 2n | 2n | 2n |
| VIII | 2n | 2n | 2n | 2n | 2n | 2n |
| IX | 3n | 3n | 3n | 3n | 3n | 6n |
| X | 2n | 2n | 2n | 2n | 2n | 2n |
| XI | 2n | 2n | 2n | 2n | 2n | 2n |
| XII | 2n | 2n | 2n | 2n | 2n | 2n |
| XIII | 2n | 2n | 2n | 2n | 2n | 2n |
| XIV | 2n | 2n | 2n | 2n | 2n | 2n |
| XV | 2n | 2n | 2n | 2n | 2n | 2n |
| XVI | 2n | 2n + small part 3n | 2n | 2n | 2n | 2n |
