# Supplementary material for: In-situ muconic acid extraction reveals sugar consumption bottleneck in a xylose-utilizing Saccharomyces cerevisiae strain
Source: Microb Cell Fact. 2021 Jun 7;20:114. doi: 10.1186/s12934-021-01594-3 (PMC8182918; doi:10.1186/s12934-021-01594-3)
Supplement: Supplementary file 7 — Additional file 7: Table S2. Common SNPs in the TN6 transformants compared to TN5. [file 12934_2021_1594_MOESM7_ESM.docx]

**Additional file 7**

**Common SNPs in the TN6 transformants compared to TN5.**

| **Gene** | **Chr.** | **Missense variant** | **TN6-1** | **TN6-2** | **TN6-3** | **TN6-4** | **TN6-5** |
| --- | --- | --- | --- | --- | --- | --- | --- |
| *CSS1* | IX | T832S |  | x | x | x | x |
| *CSS1* | IX | K831Q |  | x | x | x | x |
| *CSS1* | XVI | P830S |  | x | x | x | x |
| *CTF4* | IV | D6G |  | x | x |  |  |
| *EHD3* | IX | I469V |  | x | x |  |  |
| *FKH1* | IX | A63S |  | x | x | x |  |
| *FLO11* | IX | V863A |  | x |  | x | x |
| *FLO11* | XVI | V863L |  | x |  | x | x |
| *HXT8* | X | R319G | x |  |  | x |  |
| *KAE1* | XI | Q169E |  | x | x |  |  |
| *NUP100* | XI | S329F |  | x | x |  |  |
| *PDR15* | IV | I1525N | x | x | x | x |  |
| *PDR15* | IV | Y1526F | x | x | x | x |  |
| *PGA3* | XIII | V144A |  | x | x |  |  |
| *POG1* | IX | A207G | x | x | x | x | x |
| *QDR1* | IX | A208V | x | x | x | x | x |
| *SRB8* | III | P1044Q | x | x | x | x | x |
| *SSL2* | IX | P9S | x | x | x | x | x |
| *SUC2* | IX | V431A | x | x | x |  | x |
| *TRS120* | IV | M1055I | x |  | x | x |  |
| YIR018C-A | IX | D4Y |  | x | x | x |  |
| YNL190W | XIV | KF2NF |  |  | x | x | x |
