# Supplementary material for: In-situ muconic acid extraction reveals sugar consumption bottleneck in a xylose-utilizing Saccharomyces cerevisiae strain
Source: Microb Cell Fact. 2021 Jun 7;20:114. doi: 10.1186/s12934-021-01594-3 (PMC8182918; doi:10.1186/s12934-021-01594-3)
Supplement: Supplementary file 8 — Additional file 8: Table S3. Unique SNPs in each TN6 transformant compared to TN5. [file 12934_2021_1594_MOESM8_ESM.docx]

**Additional file 8**

**Unique SNPs in each TN6 transformant compared to TN5.**

| **Gene** | **Chr.** | **Missense variant** |
| --- | --- | --- |
| **TN6-1** | | |
| *AFT1* | VII | S200F |
| *DNF2* | IV | R437S |
| *DPH2* | XI | D355E |
| *MRD1* | XVI | E423G |
| *NTG1* | I | D399E |
| **TN6-4** | | |
| *YNL143C* | XVI | L45F |
| *YJR079W* | IV | I11F |
| **TN6-5** | | |
| *CSS1* | IX | N386S |
| *CSS1* | IX | T382S |
| *CSS1* | IX | V380E |
| *CSS1* | IX | N378T |
| *INO80* | VII | E675* |
| *JJJ2* | X | G238E |
| *JJJ2* | X | G238R |
| *ORC4* | XVI | T171I |
| *PFK26* | IX | V827F |
| *TRK2* | XI | EE449DE |
| *UBP10* | XIV | I186F |
| *UBP10* | XIV | IG186MG |
| *YIR018C-A* | IX | V11L |
| *YJR079W* | IX | N12T |
