# Supplementary material for: In-situ muconic acid extraction reveals sugar consumption bottleneck in a xylose-utilizing Saccharomyces cerevisiae strain
Source: Microb Cell Fact. 2021 Jun 7;20:114. doi: 10.1186/s12934-021-01594-3 (PMC8182918; doi:10.1186/s12934-021-01594-3)
Supplement: Supplementary file 9 — Additional file 9: Table S4. SNPs in MApw genes in the TN6 transformants compared to TN5. [file 12934_2021_1594_MOESM9_ESM.docx]

**Additional file 9**

**SNPs in MApw genes in the TN6 transformants compared to TN5.**

| **Enzyme** | **Missense variant** | **TN5** | **TN6-1** | **TN6-2** | **TN6-3** | **TN6-4** | **TN6-5** |
| --- | --- | --- | --- | --- | --- | --- | --- |
| DHSD | S61P |  |  | x |  |  |  |
| PCAD | P203S |  |  |  |  | x |  |
|  | I218V |  | x |  | x |  |  |
|  | A315T |  |  | x |  |  |  |
