# Supplementary material for: In-situ muconic acid extraction reveals sugar consumption bottleneck in a xylose-utilizing Saccharomyces cerevisiae strain
Source: Microb Cell Fact. 2021 Jun 7;20:114. doi: 10.1186/s12934-021-01594-3 (PMC8182918; doi:10.1186/s12934-021-01594-3)
Supplement: Supplementary file 10 — Additional file 10. PCA and muconic acid production by TN6-1 and TN16. (A) YP2.5%D0.5%E medium buffered with 50 mM citrate buffer and initial pH of 5.5. Strains were inoculated at OD600 4. Results are the means of two biological replicates for TN6-1 and three independent replicates for TN16. Error bars show standard deviation at each time point. (B) Total molar yield of PCA and muconic acid from fermentation experiment shown in (A) at time point 144 h. [file 12934_2021_1594_MOESM10_ESM.docx]

**Additional file 10**


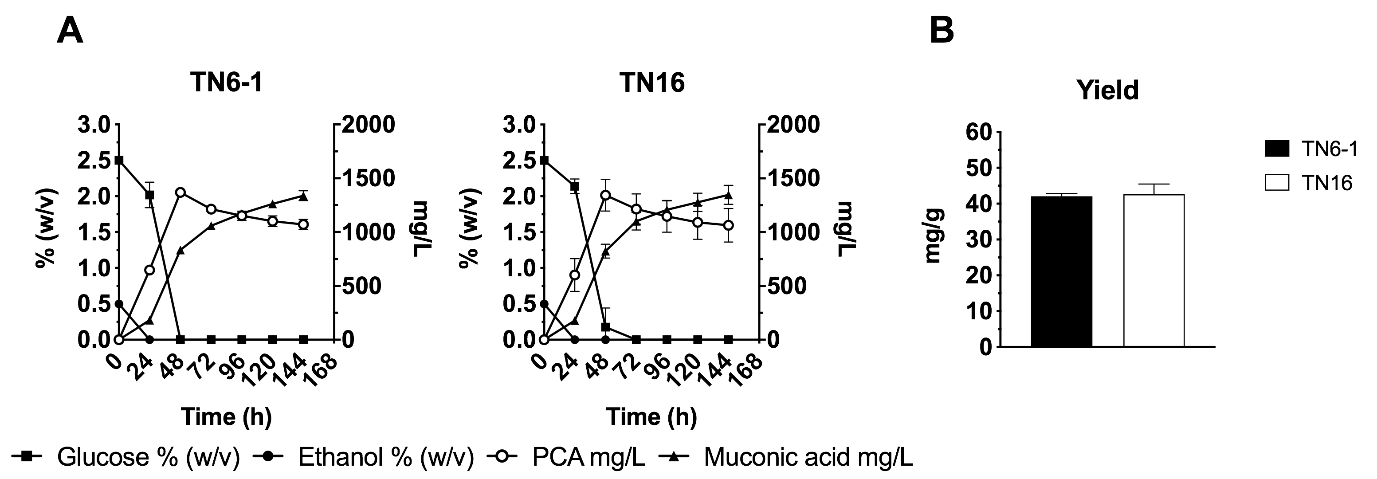


**PCA and muconic acid production by TN6-1 and TN16.**

(**A**) YP2.5%D0.5%E medium buffered with 50 mM citrate buffer and initial pH of 5.5. Strains were inoculated at OD_600_ 4. Results are the means of two biological replicates for TN6-1 and three independent replicates for TN16. Error bars show standard deviation at each time point. (**B**) Total molar yield of PCA and muconic acid from fermentation experiment shown in (**A**) at time point 144h.
