# Supplementary material for: In-situ muconic acid extraction reveals sugar consumption bottleneck in a xylose-utilizing Saccharomyces cerevisiae strain
Source: Microb Cell Fact. 2021 Jun 7;20:114. doi: 10.1186/s12934-021-01594-3 (PMC8182918; doi:10.1186/s12934-021-01594-3)
Supplement: Supplementary file 11 — Additional file 11. Comparison of different pitching rates with TN6-1. YP2%D2.25%X1.6%E medium. Results are means of two biological replicates. Error bars show standard deviation at each time point. [file 12934_2021_1594_MOESM11_ESM.docx]

**Additional file 11**

**
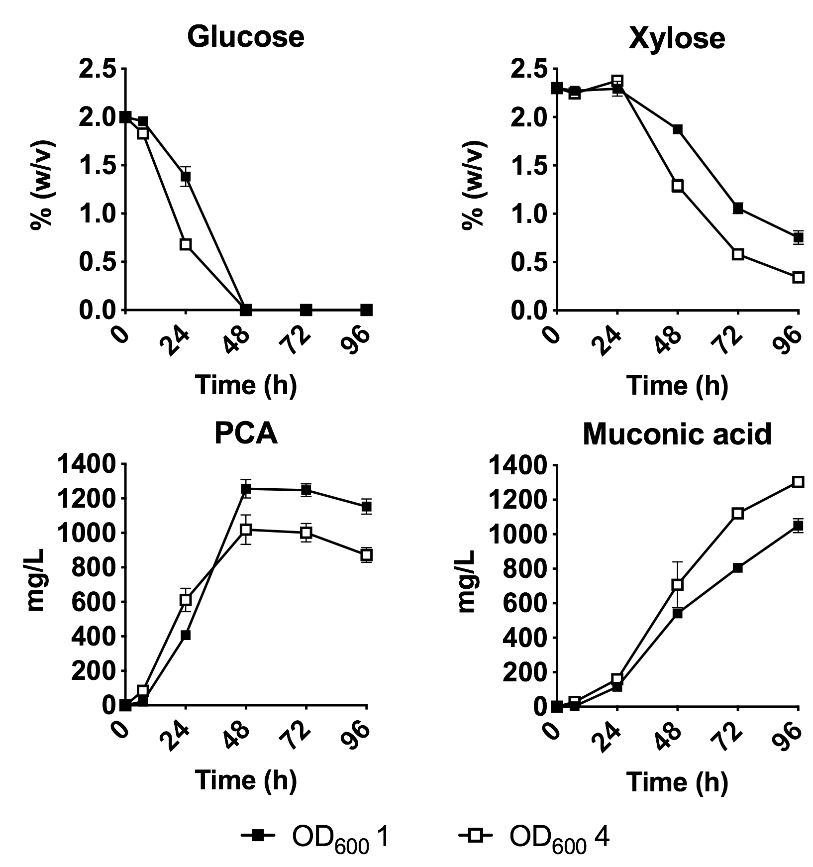
**

**Comparison of different pitching rates with TN6-1.** YP2%D2.25%X1.6%E medium. Results are means of two biological replicates. Error bars show standard deviation at each time point.
