# Supplementary material for: In-situ muconic acid extraction reveals sugar consumption bottleneck in a xylose-utilizing Saccharomyces cerevisiae strain
Source: Microb Cell Fact. 2021 Jun 7;20:114. doi: 10.1186/s12934-021-01594-3 (PMC8182918; doi:10.1186/s12934-021-01594-3)
Supplement: Supplementary file 14 — Additional file 14. Comparison of different glucose concentrations for the production of PCA and muconic acid by TN6-1 in YP medium buffered with 100 mM citrate buffer at initial pH of 5.5. YP medium with 2%, 5% or 10% glucose and 1.6% ethanol. Strains were inoculated at OD600 4. Results are means of two biological replicates. Error bars show standard deviation at each time point. [file 12934_2021_1594_MOESM14_ESM.docx]

**Additional file 14**

**
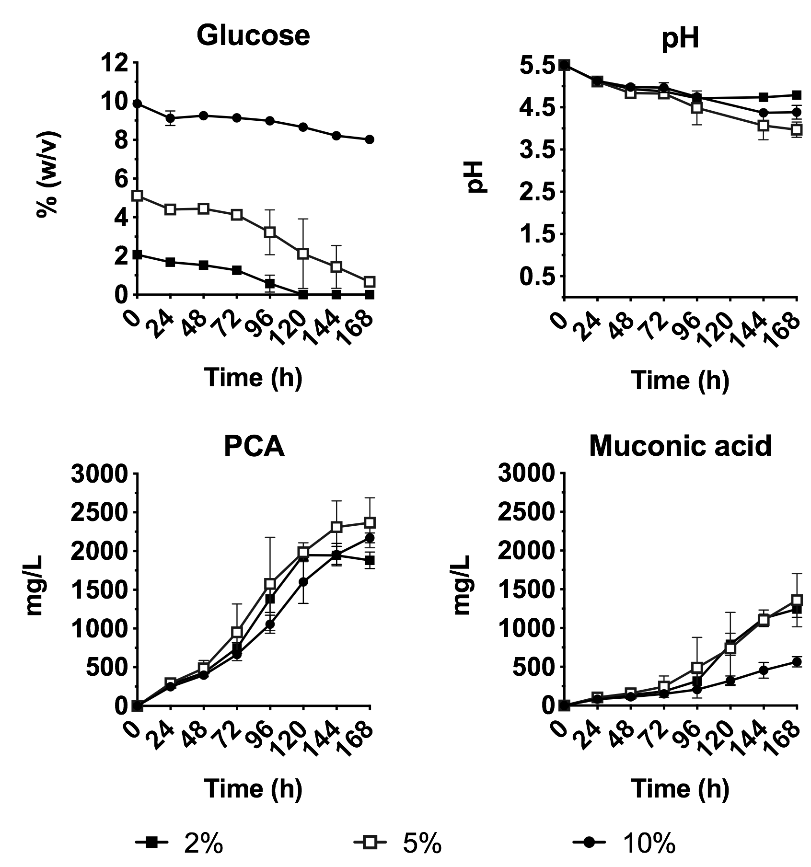
**

**Comparison of different glucose concentrations for the production of PCA and muconic acid by TN6-1 in YP medium buffered with 100 mM citrate buffer at initial pH of 5.5.** YP medium with 2%, 5% or 10% glucose and 1.6% ethanol. Strains were inoculated at OD_600_ 4. Results are means of two biological replicates. Error bars show standard deviation at each time point.
