# Supplementary material for: In-situ muconic acid extraction reveals sugar consumption bottleneck in a xylose-utilizing Saccharomyces cerevisiae strain
Source: Microb Cell Fact. 2021 Jun 7;20:114. doi: 10.1186/s12934-021-01594-3 (PMC8182918; doi:10.1186/s12934-021-01594-3)
Supplement: Supplementary file 15 — Additional file 15. Comparison of different pitching rates for the production of PCA and muconic acid with the TN6-1 strain in YP medium buffered with 50 mM citrate buffer at initial pH of 5.5. YP medium with 12% glucose and 1.6% ethanol. Results are means of two biological replicates. Error bars show standard deviation at each time point. [file 12934_2021_1594_MOESM15_ESM.docx]

**Additional file 15**

**
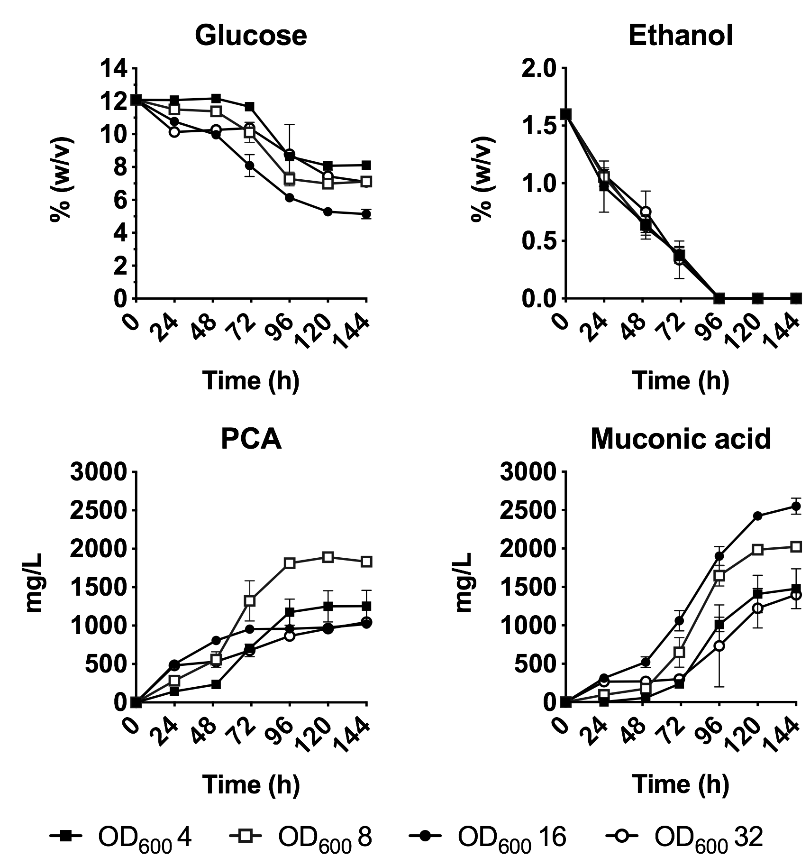
**

**Comparison of different pitching rates for the production of PCA and muconic acid with the TN6-1 strain in YP medium buffered with 50 mM citrate buffer at initial pH of 5.5.** YP medium with 12% glucose and 1.6% ethanol. Results are means of two biological replicates. Error bars show standard deviation at each time point.
