# Supplementary material for: In-situ muconic acid extraction reveals sugar consumption bottleneck in a xylose-utilizing Saccharomyces cerevisiae strain
Source: Microb Cell Fact. 2021 Jun 7;20:114. doi: 10.1186/s12934-021-01594-3 (PMC8182918; doi:10.1186/s12934-021-01594-3)
Supplement: Supplementary file 16 — Additional file 16. Determination of viability during muconic acid fermentations in YPD, YPX and YPDX. CFUs were determined at different time points during the fermentations with the TN22 strain in the absence or presence of the PPG solvent (1:5 ratio) (Fig. 7) up to 144 h. They are shown on a logarithmic scale. (At 192 h very low and highly variable CFUs were found indicating strong loss of viability after extended incubation at the end of the fermentation.) [file 12934_2021_1594_MOESM16_ESM.docx]

**Additional file 16**

**
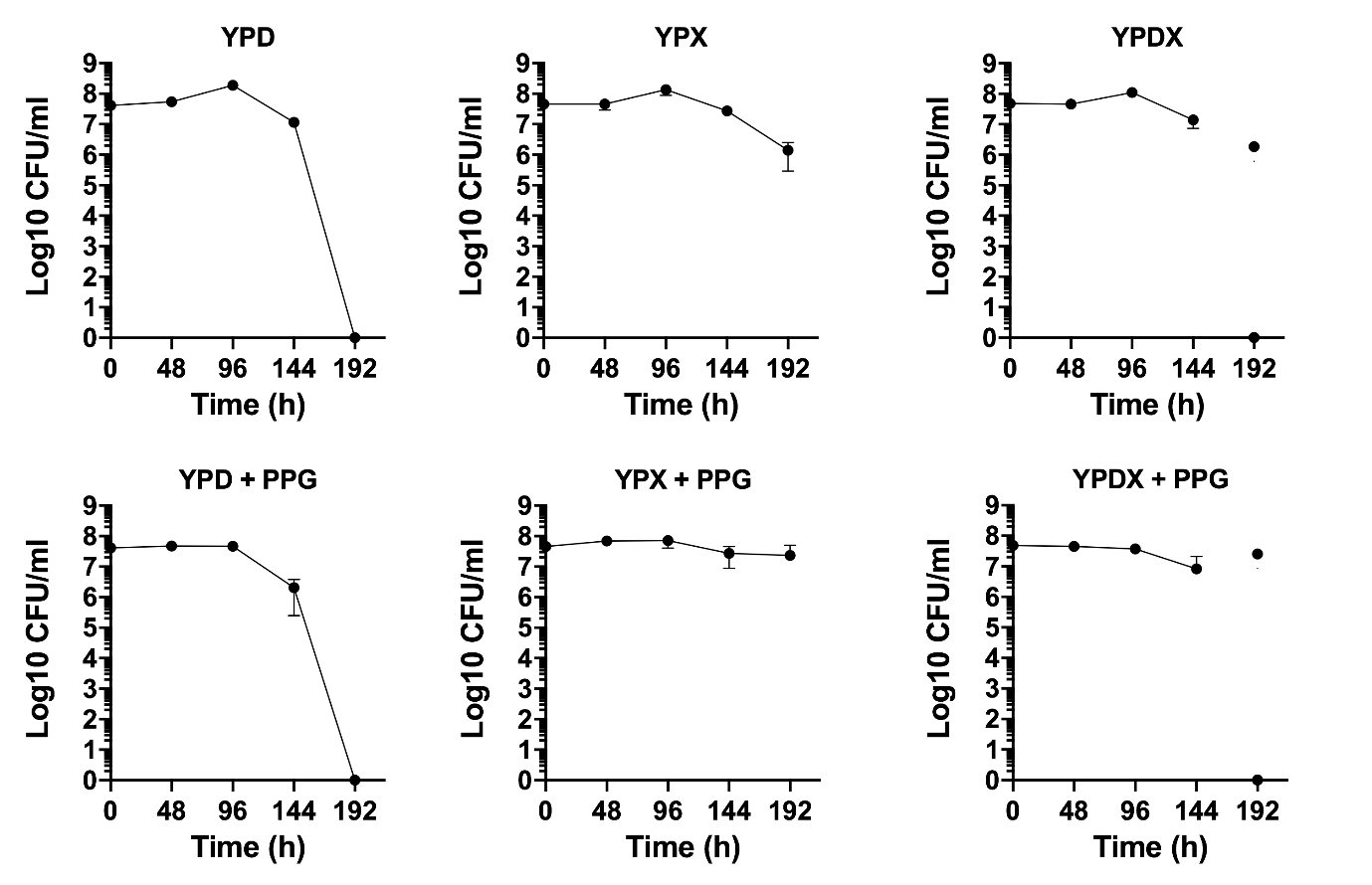
**

**Determination of viability during muconic acid fermentations in YPD, YPX and YPDX.** CFUs were determined at different time points during the fermentations with the TN22 strain in the absence or presence of the PPG solvent (1:5 ratio) (Fig. 7) up to 144h. They are shown on a logarithmic scale. (At 192h very low and highly variable CFUs were found indicating strong loss of viability after extended incubation at the end of the fermentation.)
