# Supplementary material for: In-situ muconic acid extraction reveals sugar consumption bottleneck in a xylose-utilizing Saccharomyces cerevisiae strain
Source: Microb Cell Fact. 2021 Jun 7;20:114. doi: 10.1186/s12934-021-01594-3 (PMC8182918; doi:10.1186/s12934-021-01594-3)
Supplement: Supplementary file 17 — Additional file 17. PCA and muconic acid production by the TN22 strain in the presence of the PPG solvent (1:2 ratio). YP medium containing 10% glucose (YPD), 10% xylose (YPX) or 5% glucose and 5% xylose (YPDX) with 2% ethanol, buffered with 50 mM citrate buffer at initial pH of 5.5 and inoculum OD600 16. Media contained either (A) no PPG or (B) presence of PPG (solvent/fermentation medium 1:5 ratio). (C) Muconic acid titer and yield (aqueous phase in grey plus solvent phase in white) (at 216 h) in the absence (A) and presence (B) of PPG. Muconic acid was extracted from the PPG solvent phase using 50% ethanol. Results are means of three independent replicates. Error bars show standard deviation at each time point. [file 12934_2021_1594_MOESM17_ESM.docx]

**Additional file 17**

**
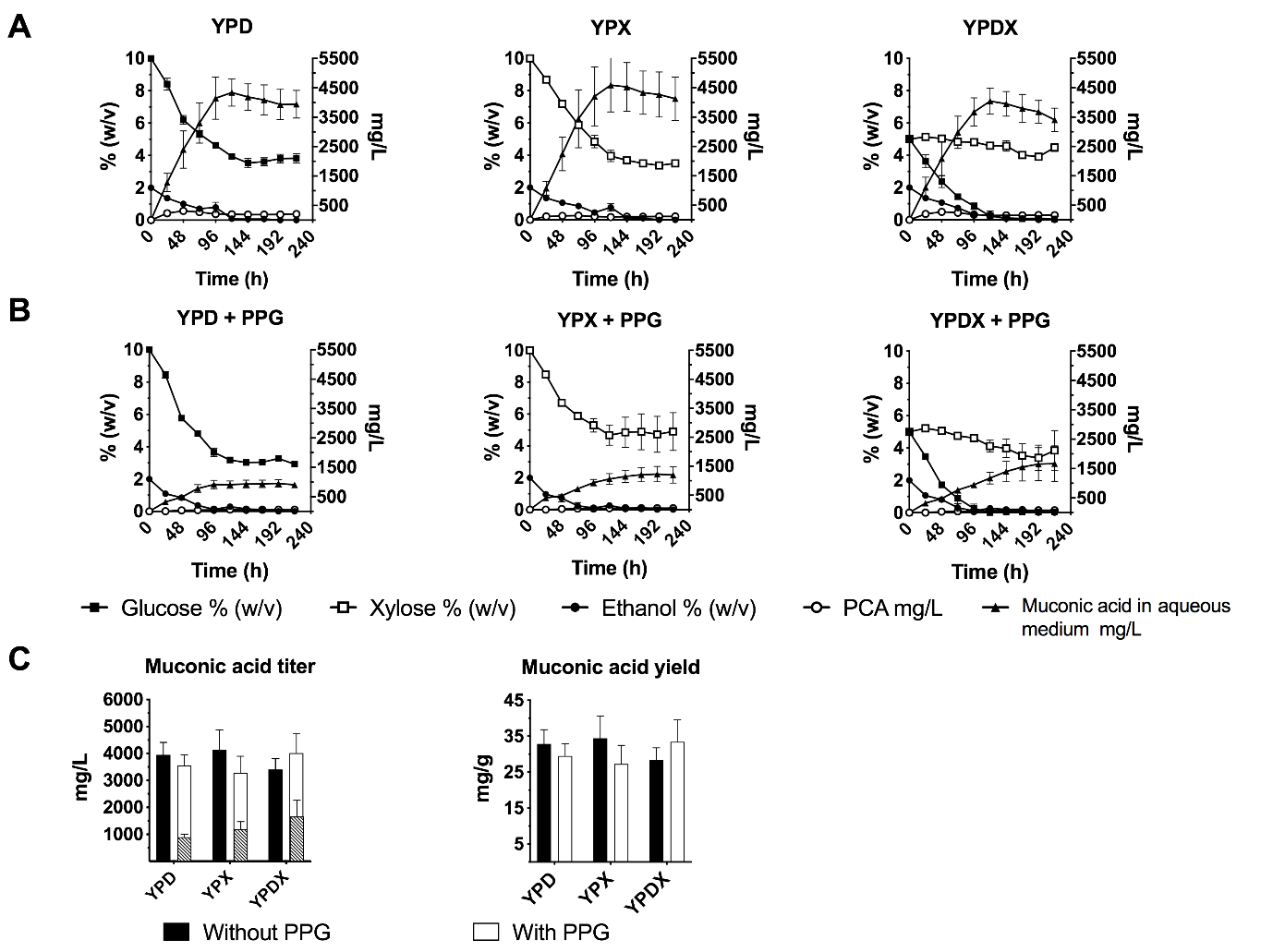
**

**PCA and muconic acid production by the TN22 strain in the presence of the PPG solvent (1:2 ratio).** YP medium containing 10% glucose (YPD), 10% xylose (YPX) or 5% glucose and 5% xylose (YPDX) with 2% ethanol, buffered with 50 mM citrate buffer at initial pH of 5.5 and inoculum OD_600_ 16. Media contained either (**A**) no PPG or (**B**) presence of PPG (solvent/fermentation medium 1:5 ratio). (**C**) Muconic acid titer and yield (aqueous phase in grey plus solvent phase in white) (at 216h) in the absence (A) and presence (B) of PPG. Muconic acid was extracted from the PPG solvent phase using 50% ethanol. Results are means of three independent replicates. Error bars show standard deviation at each time point.
