# Supplementary material for: In-situ muconic acid extraction reveals sugar consumption bottleneck in a xylose-utilizing Saccharomyces cerevisiae strain
Source: Microb Cell Fact. 2021 Jun 7;20:114. doi: 10.1186/s12934-021-01594-3 (PMC8182918; doi:10.1186/s12934-021-01594-3)
Supplement: Supplementary file 18 — Additional file 18. Determination of viability during muconic acid fermentations in YPD, YPX and YPDX. CFUs were determined at different time points during the fermentations with the TN22 strain in the absence or presence of the PPG solvent (1:2 ratio) (Additional file 17) up to 216 h. They are shown on a logarithmic scale. [file 12934_2021_1594_MOESM18_ESM.docx]

**Additional file 18**

**
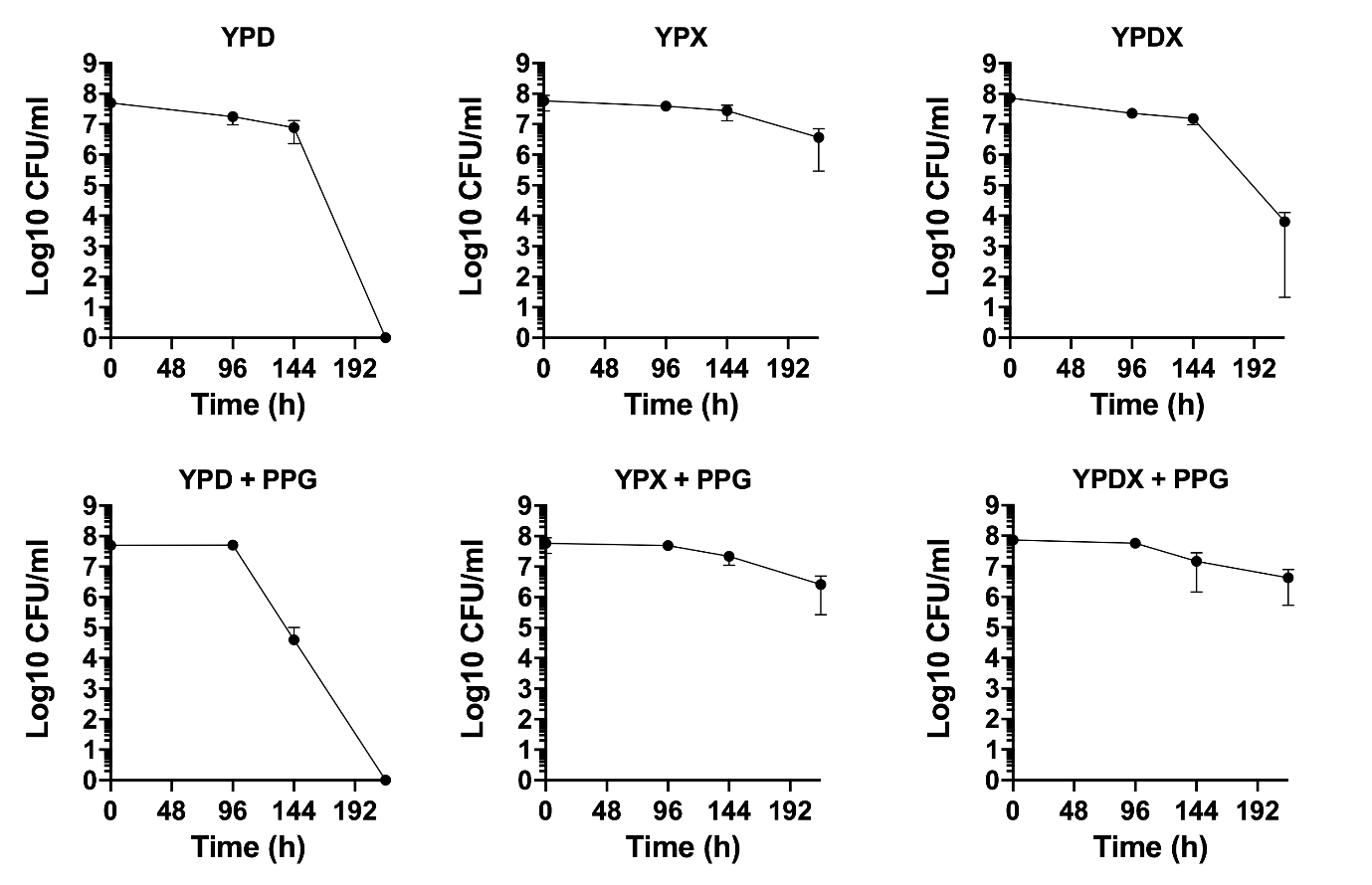
**

**Determination of viability during muconic acid fermentations in YPD, YPX and YPDX.** CFUs were determined at different time points during the fermentations with the TN22 strain in the absence or presence of the PPG solvent (1:2 ratio) (Additional file 17) up to 216h. They are shown on a logarithmic scale.
